# *Melastoma malabathricum* (L.) Smith Ethnomedicinal Uses, Chemical Constituents, and Pharmacological Properties: A Review

**DOI:** 10.1155/2012/258434

**Published:** 2011-12-28

**Authors:** S. Mohd. Joffry, N. J. Yob, M. S. Rofiee, M. M. R. Meor Mohd. Affandi, Z. Suhaili, F. Othman, A. Md. Akim, M. N. M. Desa, Z. A. Zakaria

**Affiliations:** ^1^Departments of Pharmaceutics and Pharmaceutical Sciences, Faculty of Pharmacy, Universiti Teknologi MARA, Puncak Alam Campus, Selangor, 42300 Bandar Puncak Alam, Malaysia; ^2^Faculty of Agriculture and Biotechnology, Universiti Sultan Zainal Abidin, Kampus Kota, Jalan Sultan Mahmud, 20400 Kuala Terengganu, Malaysia; ^3^Department of Biomedical Science, Faculty of Medicine and Health Sciences, Universiti Putra Malaysia, Selangor, 43400 UPM Serdang, Malaysia; ^4^Halal Products Research Institute, Universiti Putra Malaysia, Selangor, 43400 UPM Serdang, Malaysia

## Abstract

*Melastoma malabathricum* L. (Melastomataceae) is one of the 22 species found in the Southeast Asian region, including Malaysia. Considered as native to tropical and temperate Asia and the Pacific Islands, this commonly found small shrub has gained herbal status in the Malay folklore belief as well as the Indian, Chinese, and Indonesian folk medicines. Ethnopharmacologically, the leaves, shoots, barks, seeds, and roots of *M. malabathricum* have been used to treat diarrhoea, dysentery, hemorrhoids, cuts and wounds, toothache, and stomachache. Scientific findings also revealed the wide pharmacological actions of various parts of *M. malabthricum*, such as antinociceptive, anti-inflammatory, wound healing, antidiarrheal, cytotoxic, and antioxidant activities. Various types of phytochemical constituents have also been isolated and identifed from different parts of *M. malabathricum*. Thus, the aim of the present review is to present comprehensive information on ethnomedicinal uses, phytochemical constituents, and pharmacological activities of *M. malabathricum*.

## 1. Introduction

Melastomataceae plants originate in the tropic and subtropic regions, with a total of more than 4000 species in the world. In the Southeast Asian region alone, the genus *Melastoma* comprises 22 species, 2 subspecies, and 3 varieties [1]. Malaysia, particularly, with a tropical climate, is home to at least 12 species, many of which are used by natives in folk medicine. One of the plants within the Melastomataceae family that have gained herbs status in the Malay folklore belief is* Melastoma malabathricum* Linn., which has been known to comprise two subspecies, namely, *M. malabathricum *L. ssp. *malabathricum* and *M. malabathricum *Linn ssp. *normale* [2].

In general,* M. malabathricum *is a small shrub commonly found in previously cleared land, waste places, and roadside throughout the Southeast Asian countries, including Malaysia [3]. It is native to tropical and temperate Asia and the Pacific Islands [4]. The plant is one of the most common weeds that grow wildly and abundantly throughout the tropics, especially in the moist areas, and can be found in the Indian Ocean Islands, throughout South and South-East Asia, China, Taiwan, Australia, and the South Pacific Ocean [5]. Throughout Malaysia, particularly, the plant is very common in the lowland and mountain forests, chiefly in open places. *M. malabathricum *has different vernacular names depending on the location (e.g., Malaysia, Indonesia, China, and India) where the plant was found and the communities or tribes (e.g., Malay, Chinese, and Indian) that used them traditionally for medicinal purposes [4, 6–20] (Table 1).

**Figure 1:**
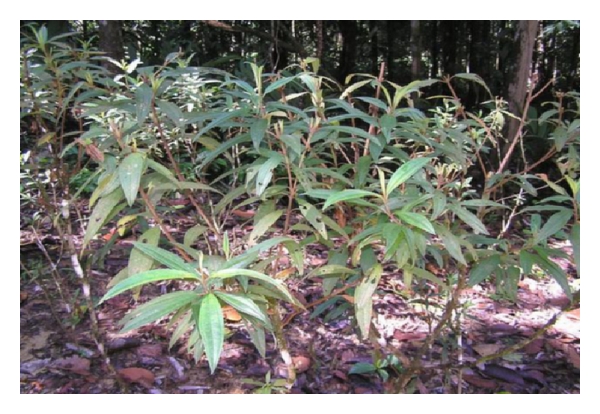
The shrubs of *Melastoma malabathricum* L. (adapted from http://www.google.com/).

**Figure 2:**
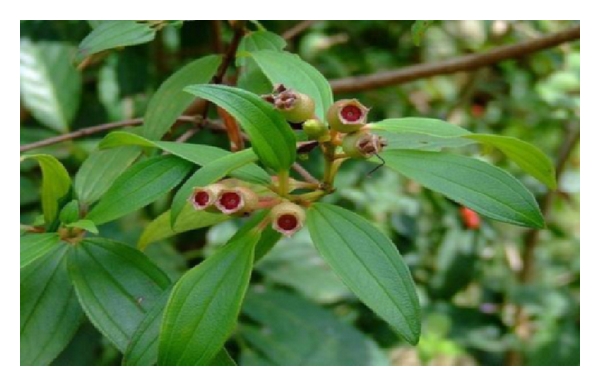
The leaves and fruits of *M. malabathricum* L. (adapted from http://www.google.com/).

**Figure 3:**
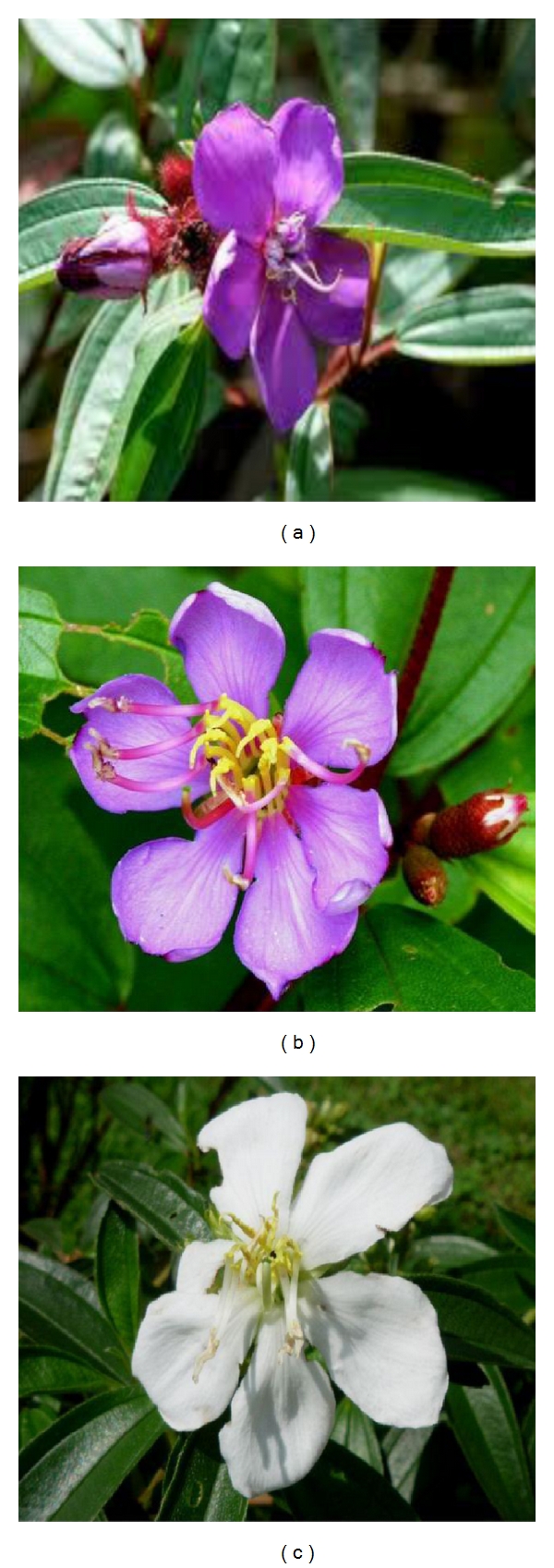
*Melastoma malabathricum *flowers with different petal colour. (a) Dark purple-magenta petals, (b) light pink-magenta petals, and (c) white petals (adapted from http://www.google.com/).

**Figure 4:**
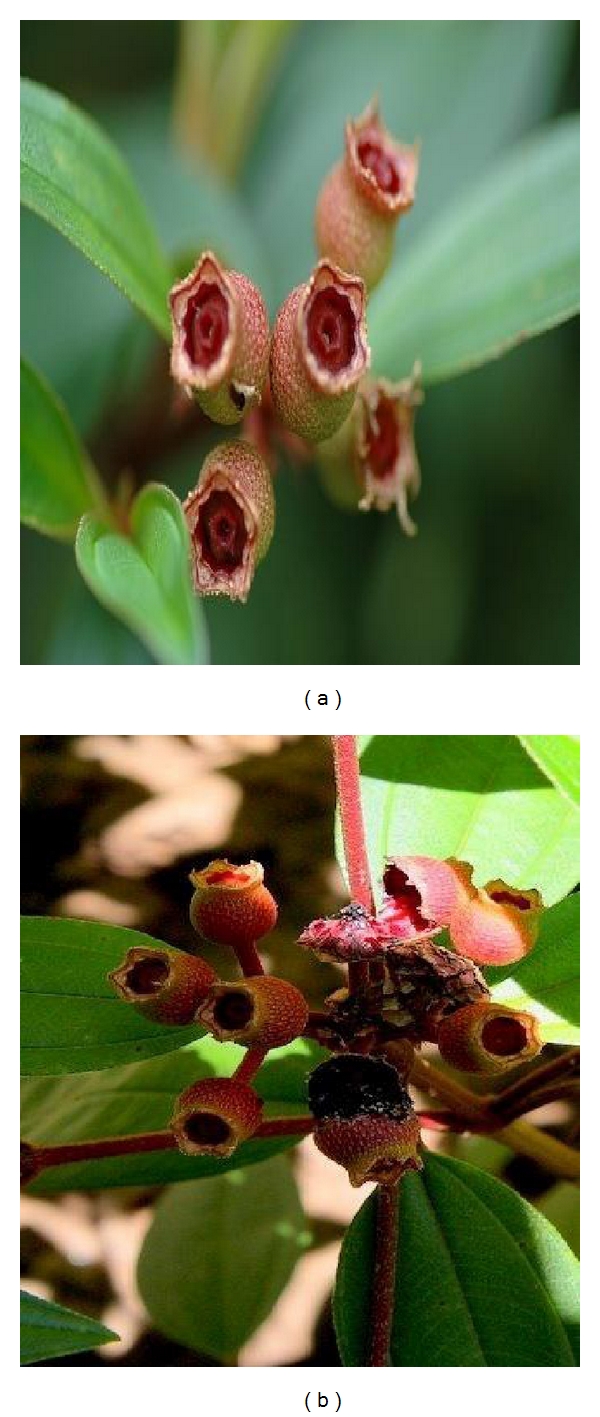
The fruits of *Melastoma malabathricum*. (a) Unripe fruits, and (b) ripe fruits revealing the soft, dark purple, sweet pulp, and numerous orange seeds (adapted from http://www.google.com/).

This showy bush of small trees rapidly colonises wastelands as their seeds are dispersed by birds. The characteristics of *M. malabathricum* include its average height of 0.5–1(−5) m high but may occasionally grow up to 5 m long (Figure 1). The stems are 4-sided to subterete, generally bristly, covered with small rough scales, and reddish. Branchlets are numerous, procumbent, densely covered with appressed scales. The petiole is approximately 0.5–1.9 cm while the leaves are blade ovate, elliptic, or elliptic-lanceolate, 4–14 × 1.7–3.5(−6) cm, stiffly papery, abaxially densely strigose and puberulous, adaxially densely strigose, secondary veins 2 (or 3) on each side of midvein, tertiary veins numerous and parallel, base rounded to subcordate, margin entire, apex acuminate (Figure 2). Inflorescences subcapitate corymbose, terminal, 3–7-flowered, with 2 leaflike bracts at base. Pedicel 2–8(−10) mm, strigose, apically 2-bracteolate, bracteoles lanceolate to subulate, 2–5 mm, abaxially densely strigose, margin ciliate. Hypanthium 5–9 mm, densely compressed strigose, margin fimbriate. Calyx lobes lanceolate to ovate-lanceolate, apex acuminate, on both sides and along their margin squamosly strigose and pubescent. Petals reddish purple, 2-3(−4) cm, margin only ciliate, apex rounded. The flowers, which are short-lived and last only a day, grow in 5 to 10 clusters and have 5 petals [21]. The flower has ten stamens of two different kinds: five larger ones with yellow filaments and purple curved upper parts including the anther and five smaller ones with yellow and straight filaments and yellow anthers. On rare occasions, *M. malabathricum* consists of three varieties, having large-, medium- and small-size flowers with dark purple-magenta petals (Figure 3(a)), light pink-magenta petals (Figure 3(b)), and (the rare variety) white petals (Figure 3(c)) [22]. The calyx closely set with short chaffy and silky or silvery scale. Longer stamens with connective long extended at base, curved, apex bifid. Shorter stamens with anthers 2-tuberculate at base; connective not extended. Ovary half inferior, densely strigose, apically with a ring of setae. Fruit urceolate-globular, 6–15 × 6–12 mm, succulent, densely squamose strigose [10]. The fruits are technically classified as berries, and, when they are ripe, they break open irregularly to reveal the soft, dark purple, sweet but rather astringent-tasting pulp and numerous orange seeds (Figures 4(a) and 4(b)). The seeds are dimorphic: with or without embryos. Fertile seeds are folded or spiral, triangular to D-shaped in outline, 0.45–0.8 mm long, 0.35–0.6 mm wide, 0.17–0.3 mm thick, with light yellow or pale to dark cream-coloured testa. Seeds without embryo are similar to the fertile seeds but smaller, 0.3–0.5 mm long, 0.2–0.3 mm wide, 0.2 mm thick, appear collapsed, dented, or wrinkled and with completely black or reddish-black testa. The seeds are tasteless and can be eaten, and they stain the tongue black. The name “melastoma” is Greek for “black mouth,” a name appreciated by generations of children who have eaten the berries. *M. malabathricum* has evergreens and flowers throughout the year [23].

*M. malabathricum *has been claimed to possess various medicinal values according to the communities/tribes traditional beliefs and, interestingly, the whole plant could be used as herbal medicine. It is also a well-known herb in Malaysia, particularly, where its leaves, shoots, and roots are prepared in various ways for treatment of different diseases and ailments (Table 2(a)). Many reviews have appeared in the literature regarding *M. malabathricum* medicinal uses. However, none have described the complete chemical and pharmacological properties of this important ethnomedicinal plant. Therefore, we aimed to compile an up-to-date and comprehensive review of *M. malabthricum* that covers its ethnomedicinal uses, phytochemical contents, and scientifically proven pharmacological properties.

## 2. Ethnomedicinal Uses

There are a lot of uses for *M. malabathricum* reported in folk medicine, but not supported by clinical data [23–25]. Generally, various parts (e.g., leaf, roots, and/or barks) of the plant are used in Malay, Indian, and Indonesian folk medicines to treat various types of ailments and diseases, for example, diarrhoea, dysentery, leucorrhoea, hemorrhoids, cuts and wounds, infection during confinement, toothache, stomachache, flatulence, sore legs, and thrush [23, 26, 27] (Table 2). There is also report on the use of *M. malabathricum *seeds in the famous “poh chi” pills to treat diarrhea in traditional Chinese medicine [28].

### 2.1. Reports on Traditional Uses of Various Parts of *M. malabathricum*

The leaves are chewed up, pounded, and applied as paste on cuts or wounds or finely chopped up and squeezed to apply the juice onto the wound to stop bleeding [29, 30]. According to Sharma et al. [11] the leaves can also be used to prevent scarring from smallpox, to treat dysentery, diarrhoea, and piles, and as a tonic. The young leaves are eaten to treat diarrhea while the young premature leaves are consumed raw to cure dysentery [23, 31]. The shoots can be ingested to treat puerperal infections, high blood pressure, and diabetes [23, 24] while the shoots juice can also be used as a mouthwash to relieve a toothache or to treat leukorrhea. Other than those mentioned above, the leaves are also medicinally useful to treat ulcers, gastric ulcers, scar, pimple, and black spot at skin [32]. The roots can also be used as mouthwash to relieve a toothache and to treat epilepsy [24, 25, 32], given to postpartum women to aid healing and womb strengthening [7, 25, 30] or to alleviate rheumatism, arthritis, and tenderness in the legs [23, 24]. The decoction of the roots is used to treat diarrhea [33]. In addition, the roots' liquid can be applied to lessen the soreness due to thrush in children [23, 24]. The barks are medicinally useful for the treatment of various skin diseases [34]. The flowers are also used in India to treat cancer [35].

Other than that, the powdered leaves and roots can be applied to wounds and pox scars to aid the healing process [7, 24] or used to relieve the discomfort of hemorrhoids [7] with the former also used as astringent for dysentery [9]. The juice of leaves and roots is used as a digestive aid [9]. Furthermore, the leaves and flowers are useful for the treatment of cholera, diarrhoea, prolonged fever, dysentery, leucorrhoea, wounds, and skin diseases and for the preparation of gargles [11, 23, 24, 36]. The decoction of roots and leaves or roots alone are also traditionally used to tone up the uterus after childbirth in order to strengthen the womb and accelerate wound healing. Other than that, women also use this herb for excessive menstrual bleeding and cramps, to relieve postmenstrual syndrome, stomach ache, and white discharge, and to enhance fertility [23]. Its flowers, seeds, and leaves are used to reduce white vaginal discharge and indigestion [9]. The flowers of *M. malabathricum* are also used as a nervous sedative and for hemorrhoidal bleeding [9]. The combination of leaves and flowers is used as astringent in leukorrhea and chronic diarrhea [9]. Despite being a traditional medicinal herb that is widely used, particularly, in Malay culture, there is not much scientific study carried out on *M. malabathricum*.

### 2.2. Reports on the Uses of *M. malabathricum* by Various Communities/Tribes

The list of medicinal uses of *M. malabathricum *according to the different communities or tribes reported around the world is shown in Table 2(b). According to Elliott and Brimacombe [37] the cold infusion of *M. malabthricum *flowers is an optional ingredient added to an oral remedy for anaemia associated with gastrointestinal bleeding and epigastric pain. The Talang Mamak peoples of Riau province, Sumatra, Indonesia, used the ground leaf and applied it as a compress to cuts and wounds [8]. A survey of the Malay ethnomedico botany in the Machang district, Kelantan, Malaysia, revealed the application of *M. malabathricum* fruits juice on dry lip [38] while in the Gemenceh district, Negri Sembilan, Malaysia, the Malays applied *M. malabathricum* pounded leaves onto wounds to accelerate healing [39]. The Jah Hut tribe in Jerantut district, Pahang, Peninsular, Malaysia, used the roots of *M. malabathricum* as decoction to treat diarrhea [33]. The native peoples of Mizoram, India, used the decoction of the leaf or its juice, which is taken orally, in the treatment of diarrhoea and dysentery [11]. The Meitei community living in Manipur district in India and other tribes living in the Similipal Biosphere Reserve, situated in Mayurbhanj district of Orissa, India, used the bark and leaves of *M. malabathricum *for treating skin troubles, leukorrhea, diarrhea, and dysentery [14] while the Didayi tribe of Malkangiri district of Orissa, India, used the leaf externally as paste to treat cuts and wounds [13]. The Sundanese community of the West Java, Indonesia, uses the leaf of *M. malabathricum* as topical application or oral ingestion to treat toothache and for postpartum remedy [40]. The Marmas community of Naikhongcharri, Bandarban district, Bangladesh, used the root juice to treat jaundice [18]. The Garo tribal community living in Netrakona district, Bangladesh, used the leaf juice as diuretic and to treat various urinary problems [19]. The Murong tribes residing in the Rangamati district in the Chittagong Hill Tracts region, Bangladesh, used the squeezed juice from the roots or water extract of the boiled roots orally to treat leukorrhea [20]. In Tahiti, *M. malabathricum* is used to treat diarrhea and dysentery with its bark decoction used as gargle [9]. The Naga tribe of Manipur district, India, used the fresh and dry leaves of *M. malabathricum* to treat cuts and wounds, stomach disorder, and fever [15].

## 3. Phytochemical Constituents

Various phytochemical groups and constituents have been identified in *M. malabathricum *since 1968 and are strongly associated with its ethnomedicinal values (Tables 3(a) and 3(b)). Earlier study by Lowry [41] showed the presence of ellagic acid and anthocyanin (e.g., malvidin-3,5-diglucoside) in the methanol extract of *M. malabathricum* barks (MMMBk) and aqueous extract of *M. malabathricum* flowers (AMMFw), respectively. Meanwhile, Lowry [42] also reported the presence of anthocyanins (e.g., cyanidin- (Cy-) 3-glucoside and Cy-3,5-diglucoside) in the water extract of *M. malabathricum *fruits (WMMFr). Manzoor-I-Khuda et al. [43] reported the isolation of *β*-sitosterol and a triterpenoid designated as melastomic acid (5-hydroxylup-20(29)-en-28-oic acid) from the ethanolic extract of *M. malabathricum* roots (EMMR). Dinda and Saha [44] reported the isolation of 1-octyl docosanoate and 11-methyl-1-tricontanol while Dinda and Saha [45] reported the presence of fatty acids and sterols. Das and Kotoky [46] reported the isolation of a new aliphatic constituent, namely, 32-methyl-1-tritriacontanol, together with ursolic acid, p-hydroxybenzoic acid and gallic acid, and kaempferol from the leaves and flowers of *M. malabathricum*. Compounds like kaempferol-3*-O-β*-D-xyloside, quercetin-3*-O-α*-L-rhamnosyl-(1→2)-*β*-D-galactoside, flavan-3-ol, 4′-methylpeonidin-7*-O-β*-D-glucoside, anthocyanins, and tannins have also been isolated from the aerial part of *M. malabathricum* [35, 47].

Yoshida et al. [48] reported the isolation of isoquercitrin 6′′-0-gallate, a flavonoid glycoside, three new dimeric hydrolysable tannins, namely, malabathrins B, C, and D, and the eleven known hydrolysable tannins from the 70% acetone extract of *M. malabathricum* leaves (AcMML). Among the eleven known tannins, seven were monomeric hydrolysable tannins, namely, 1,4,6-tri*-O-*galloyl-*β*-D-glucoside, 1,2,4,6-tetra*-O-*galloyl-*β*-D-glucoside, strictinin, casuarictin, pedunculagin, nobotanin D, and pterocarinin C while the other four were hydrolysable tannin oligomers which were identified as nobotanins B, G, and H (dimers) and nobotanin J (trimer). In the same year, Yoshida et al. [49] successfully isolated malabathrins A, E, and F, new complex tannins in which an ellagitannin and a flavan-3-ol are bound by a C-glycosidic linkage belonging to type II+ tannins, and other tannins, namely, casuarinin, (-)-epicatechin gallate, (-)-epicatechin, stachyurin, procyanidin B-5 and B-2, stenophyllanins A and B, alienanin B, and brevifolincarboxylic acid. The chromatographic separation of the hexane part of the methanolic extract of *M. malabathricum *aerial parts (MMMAp) led to the identification of stearic acid, *β*-sitosterol and ursolic acid [50]. On the other hand, Nurestri et al. [51] have successfully isolated three pentacyclic triterpenoids, namely, ursolic acid, 2-hydroxyursolic acid and asiatic acid, as well as glycerol-1,2-dilinolenyl-3*-O-β*-D-galactopyranoside and glycerol 1,2-dilinolenyl- 3*-O-*(4,6-di*-O-*isopropylidene)-*β*-D-galactopyranoside from the methanolic extract of *M. malabathricum *leaves (MMML) with light pink-magenta petals [51]. Nurestri et al. [51] also isolated *β*-sitosterol, *α*-amyrin, uvaol, quercetin, quercitrin, rutin, and sitosterol-3*-O-β*-D-glucopyranoside from the hexane fraction of MMML. Ali et al. [52] isolated three urs-12-ene pentacyclic triterpenoids, namely, ursolic acid, 2*α*-hydroxyursolic acid and asiatic acid, *β*-sitosterol 3*-O-β*-Dglucopyranoside, glycerol 1,2-dilinolenyl-3*-O-β*-D-galactopyranoside and glycerol 1,2-dilinolenyl-3*-O-*(4,6*-O-*isopropylidene)-*β*-D-galactopyranoside from the 90% aqueous methanolic extracts of *M. malabathricum* fresh leaves (AMMML). In addition, subjection of the ethyl acetate-soluble part of an 90% aqueous methanolic extract of *M. malabathricum* flowers (AMMMFw) to isolation and identification of bioactive compounds yielded ellagic acid and six flavonoids which were identified as quercetin, kaempferol, kaempferol 3*-O-α*-L-rhamnopyranoside, kaempferol 3*-O-β*-D-glucopyranoside, kaempferol 3*-O-β*-D-galactopyranoside, and kaempferol 3*-O-*(2′′,6′′-di*-O-*E*-p-*coumaryl)-*β*-D-galactopyranoside [53, 54]. Koay [23] also cited that the n-hexane (HMML), ethyl acetate (EAMML), and MMML extracts of *M. malabathricum *leaves yielded three new compounds, 2,5,6-trihydroxynaphtoic carbonic acid, methyl-2,5,6-trihydroxynaphtalene carbonate, and flavonol glycoside derivative. The n-hexane extract of *M. malabathricum* roots (HMMR) contained betulinic acid, serrat-14-en-16-one, and 2-(2′-hydroxyvinyl)-1-methyl-4-propoxyphthalate. The ethyl acetate extract of *M. malabathricum* flowers (EAMMFw) yielded three compounds, kaempferol-3*-O-β*-D-glucoside, kaempferol, and naringenin, while the methanol extract of *M. malabathricum* flowers (MMMFw) was found to contain kaempferol-3*-O-*(2′′,6′′-di*-O-p-trans*-coumaroyl)-glucoside and kaempferol-3*-O-β*-D-glucoside. The ethyl acetate extract of *M. malabathricum* fruits (EAMMFr) afforded betulinic acid, while the n-hexane extract of the stems produced *α*-amyrin. Nazlina et al. [55] successfully isolated rutin, quercitrin, and quercetin from the MMML using TLC assay. In addition, Susanti et al. [56] also reported the isolation of a triterpene (*α*-amyrin) and two amides (patriscabatrine and auranamide) from the HMML, two flavonoids (quercetin and quercitrin) from the EAMML, and two flavonoids (quercitrin and kaempferol-3*-O-*(2′′,6′′-di*-O-p-trans*-coumaroyl)-*β*-glucoside) from the MMML after successive extraction of the leaves of *M. malabathricum* with white petals. Zakaria et al. [30] reported the presence of flavonoids, triterpenes, tannins, saponins, and steroids, but no alkaloids in the leaves of *M. malabathricum *found in Malaysia. Simanjuntak [57] also reported the presence of flavonoids, saponins, tannins, glycosides, and steroids/triterpenoids in the leaves of *M. malabathricum* collected in Sumatera, Indonesia. Faravani [58] identified several secondary metabolites from the MMML and methanol extract of *M. malabathricum* roots (MMMR), such as hexacosanoic acid, gallic acid, flavonoids and flavonoids glycosides, phenolics, triterpenes, tannins, saponins, and steroids. Further analyses of the MMML yielded 3 urs-12-ene pentacyclic triterpenoids, namely, ursolic acid, 2**α**-hydroxyursolic acid and asiatic acid, *β-*sitosterol 3*-O-β*-D-glucopyranoside, glycerol 1,2-dilinolenyl-3*-O-*β**-D-galactopyranoside, and glycerol 1,2-dilinolenyl-3*-O-*(4,6*-O-*isopropylidene)-**β**-D-galactopyranoside. On the other hand, ethyl acetate soluble part of AMMMFw contains a host of compounds, namely, ellagic acid and 6 flavonoids, namely, quercetin, kaempferol, kaempferol 3*-O-α*-Lrhamnopyranoside, kaempferol 3*-O-β*-D-glucopyranoside, kaempferol 3*-O-β*-D-galactopyranoside, and kaempferol 3*-O-*(2′′,6′′-di*-O-E-p-*coumaryl)-*β*-D-galactopyranoside [53, 54]. Lohézic-Le Dévéhat et al. [32] have reported the presence of flavonoids and hydrolysable tannins in the aerial parts of *M. malabathricum* following the preliminary phytochemical screening.

In addition, Dinda and Saha [59] and Yeoh et al. [60] have also reported on the presence and total leaf amino acid compositions of *M. malabathricum*. The leaf of *M. malabathricum* was found to contain all important amino acids, namely, Asp, Thr, Ser, Glu, Pro, Gly, Ala, Val, Met, lie, Leu, Tyr, Phe, His, Lys, Trp, and Arg with their respective percentage of total amino acids of 11.5, 5.5, 5.1, 13.5, 6.2, 5.4, 6.4, 4.9, 2.3, 3.6, 9.9, 5.2, 6.2, 2.3, 6.9, 0.1, 4.9, and 4.24.

## 4. Pharmacological Activities

Various parts of *M. malabathricum* have been claimed to possess medicinal values, which is supported particularly by the Malay and Indian traditional uses of the plants in the treatment of a number of diseases as described earlier. Scientifically, *M. malabathricum*, prepared as extracts using different types of solvents and tested using a range of *in vitro *and *in vivo *test models, demonstrated various pharmacological potentials that required in-depth studies (Table 4). The plant, regardless of the parts used, has been shown to exert antibacterial, antiviral, antiparasitic, antioxidant, cytotoxicity, anticoagulant, platelet-activating factor inhibitory, wound healing, antiulcer, antidiarrheal, antivenom, anti-inflammatory, antinociceptive, and antipyretic activities at different doses/concentrations. The following subchapters will discuss in detail those scientific findings related to pharmacological properties of *M. malabathricum*.

### 4.1. Acute Toxicity Study

Sunilson et al. [61] have determined the acute toxicity LD_50_ level of water extract of *M. malabathricum* leaves (WMML) collected in the State of Selangor, Malaysia. The extract, in the doses of 62.5, 125, 250, 500, 1000, and 2000 mg/kg, was administered orally in mice. The authors recorded general signs and symptoms of toxicity, intake of food and water, and mortality for 48 h. The acute toxicity study showed that the WMML administered up to 2000 mg/kg dose to the mice showed neither mortality nor any visible clinical signs of general weakness in the animals. This indicates that the WMML is safe for consumption even at the highest dosage (2000 mg/kg) tested.

### 4.2. Antibacterial Activity

Grosvenor et al. [62] studied the antimicrobial property of 70% methanol extract of combined *M. malabathricum* leaf, stem, and flower (MMMClsf), collected from the Riau province, Sumatra, Indonesia, against *Escherichia coli*, *Staphylococcus aureus*, *Saccharomyces cerevisiae*, and *Fusarium oxysporum *using the agar diffusion assay. The extract was effective only against *S. aureus*, *S. cerevisiae*, and *F. oxysporum*. Standard antimicrobial references used were 1 mg/mL chloramphenicol, 1 mg/mL tetracycline, and 10000 units/mL nystatin suspensions. The chloramphenicol and tetracycline exerted antimicrobial activity against *S. aureus *and *E. coli*, while nystatin suspension exhibited antimicrobial activity against *S. cerevisiae *and* F. oxysporum*.

Wiart et al. [63] studied the antibacterial and antifungal properties of MMML, collected from Kuala Kangsar in the State of Perak, Malaysia, against *Bacillus cereus, B. subtilis*, *Escherichia coli*, *Pseudomonas aeruginosa *and* Candida albicans* using the disc diffusion method. Standard antibiotics used were 10 *μ*g gentamycin and 20 *μ*g nystatin for antibacterial and antifungal comparison, respectively. The 1000 *μ*g MMML exerted poor antimicrobial activity with zone of growth inhibition recorded at 7 mm only against *B. subtilis*. In comparison, gentamycin was effective against all microorganisms tested, except *C. albicans*, with zone of inhibition ranging between 14 and 20 mm while nystatin was effective against *C. albicans* with zone of inhibition recorded at 11 mm.

Thatoi et al. [14] reported the antimicrobial activity of the aqueous extract of different parts of *M. malabathricum* (e.g., AMML, stem (AMMS), bark (AMMBk), bulb (AMMBb), fruit (AMMFr), and root (AMMR)), collected from Simlipal Biosphere Reserve, Orissa, India, against a panel of Gram-positive and Gram-negative human pathogenic bacteria (e.g., *Staphylococcus aureus* MTCC 1144, *B. licheniformis *MTCC 7425, *B. brevis* MTCC 7404, *B. subtilis *MTCC 7164, *S. epidermidis* MTCC 3615, *Streptococcus aureus* (lab isolate), *P. aeruginosa *MTCC 1034, *E. coli *MTCC 1089, *Vibrio cholerae* (lab isolate), and *Shigella flexneri *(lab isolate)) and a fungal (e.g., *C. krusei *(lab isolate)). All extracts, in the volume of 200 *μ*L and in the concentration of 200 mg/mL each, were tested using the agar cup method. From the results obtained, AMML was considered to have an outstanding antimicrobial activity as indicated by the inhibition zones produced that are more than 20 mm. The AMML was effective against *B. brevis*, *V. cholerae*, *C. krusei*, and *B. subtilis*, while the AMMBk was effective against *S. aureus*, *B. brevis*, and* V. cholerae*.

Johnny et al. [64] reported the antifungal activity of MMML, AcMML, and chloroform (CMML) extract of *M. malabathricum* leaves collected from Sarikei in the State of Sarawak, Malaysia, against *Colletotrichum gloeosporioides*, a plant pathogenic fungus isolated from mango. The antifungal activity was tested using agar-disc dilution assay followed by determination of minimum inhibition concentration (MIC) and the rate of sporulation assay. Benomyl was used as positive control. *M. malabathricum* extracts showed 40–55% antifungal activity against *C. gloeosporioides *at varying concentrations. The MMML exhibited antifungal activity of 53.09 ± 0.75 mm at 10.00 *μ*g/mL, 48.85 ± 0.85 mm at 1.00 *μ*g/mL, 44.94 ± 0.66 mm at 0.10 *μ*g/mL, and 44.12 ± 0.45 mm at 0.01 *μ*g/mL against *C. gloeosporioides*, while the CMML exhibited antifungal activity of 49.45 ± 0.32 mm at 10.00 *μ*g/mL, 46.50 ± 0.84 mm at 1.00 *μ*g/mL, 42.55 ± 0.71 mm at 0.10 *μ*g/mL, and 41.33 ± 0.51 mm at 0.01 *μ*g/mL against *C. gloeosporioides*. On the other hand, the AcMML exhibited antifungal activity of 48.54 ± 0.41 mm at 10.00 *μ*g/mL, 43.74 ± 1.11 mm at 1.00 *μ*g/mL, 39.43 ± 0.96 mm at 0.10 *μ*g/mL, and 37.75 ± 0.56 mm at 0.01 *μ*g/mL against *C. gloeosporioides*. The MIC value recorded for the extracts was 20.00 *μ*g/mL. In the next studies to determine the inhibition of sporulation of *C. gloeosporioides*, the MMML exerted inhibition of sporulation (×10^5^) of 2.33 ± 0.04 at 10.00 *μ*g/mL, 2.29 ± 0.03 at 1.00 *μ*g/mL, 2.20 ± 0.03 at 0.10 *μ*g/mL, and 1.98 ± 0.03 at 0.01 *μ*g/mL against *C. gloeosporioides*, while the CMML exerted inhibition of sporulation (×10^5^) of 2.27 ± 0.04 at 10.00 *μ*g/mL, 1.89 ± 0.03 at 1.00 *μ*g/mL, 1.80 ± 0.03 at 0.10 *μ*g/mL, and 1.77 ± 0.03 at 0.01 *μ*g/mL against *C. gloeosporioides*. On the other hand, the AcMML exerted inhibition of sporulation (×10^5^) of 2.24 ± 0.04 at 10.00 *μ*g/mL at 10.00 *μ*g/mL against 1.76 ± 0.03 at 1.00 *μ*g/mL, 1.75 ± 0.02 at 0.10 *μ*g/mL, and 1.76 ± 0.03 at 0.01 *μ*g/mL against *C. gloeosporioides*. 

Maji et al. [65] examined the antimicrobial efficiency of WMML, AcMML, and benzene (BMML) extracts of *M. malabathricum* leaves collected from Gurguripal forest, Midnapur, West Bengal, India, against seven human pathogens (e.g., *E. coli *(multi-drug-resistant (MDR)), *S. aureus *(MDR), *K. pneumoniae, B. cereus, V. cholera*, and *C. albicans*) using the agar well diffusion method with Ciprofloxacin (50 *μ*g/mL) used as standard antibiotic. *M. malabathricum*, in the volume of 50 *μ*L/well and in the concentration of 1.00 mg/mL, was considered to possess moderate antimicrobial activity as indicated by its ability to produce zone of inhibition ranging between 8 to 11 mm against all pathogenic microorganisms. In comparison, Ciprofloxacin produced the zone of inhibition ranging between 14 and 27 mm. The MIC value for the WMML, BMML and AcMML against *V. cholerae* was 0.65, 0.80 and 0.80 mg/mL while the MIC value for the WMML and AcMML against *S. aureus* (MDR) was 0.80 and 0.79, respectively. For *E. coli* (MDR), *K. pneumoniae, C. albicans *and *B. cereus*, only the AcMML produced MIC value, which was recorded at 0.62, 0.79, 0.80 and 0.80 mg/mL, respectively. In term of the MBC value, the three extracts of *M. malabathricum* caused bactericidal activity against *V. cholera *at the concentration of 0.90 mg/mL while the AcMML and BMML exerted bactericidal effect against *S. aureus* (MDR) at 1.00 mg/mL. For *E. coli* (MDR), *K. pneumoniae*, *C. albicans* and *B. cereus*, the MBC value was recorded only for the AcMML extract, which is at 0.70, 0.95, 0.90 and 0.90 mg/mL, respectively. However, the MIC and MBC value for Ciprofloxacin was not determined.

In an attempt to study the wound healing properties of the leaves of white *M. malabathricum* collected from the State of Selangor, Malaysia, Sunilson et al. [66] also carried out antibacterial study on the MMML against the four clinical isolates (A, B, C, and D) of *S*. *aureus *and 3 clinical isolates (A, B, and C) of *P*.* aeruginosa *obtained from sores of different patients using a modification of the agar well diffusion technique. Based on the data obtained, the extract, which was tested in the concentrations of 0.5, 1.0, 2.0, 3.0, 4.0, 6.0, 8.0, and 16.0 mg/mL, exhibited antibacterial activity at the MIC value of 3.0 mg/mL for A, B, and D and 7.0 mg/mL for C clinical strains of *S. aureus*, respectively. On the other hand, the MIC value recorded for the three clinical isolates of *P. aeruginosa* was 8.0 mg/mL. However, no standard antibiotics were used as references.

### 4.3. Antiviral Activity

Lohézic-Le Dévéhat et al. [32] investigated the antiviral activity of ten methanolic extracts from eight Indonesian medicinal plants, which included *M. malabathricum*, against HSV-1 and Poliovirus. The virus titre was estimated from cytopathogenicity and expressed as 50% tissue culture infectious doses per millilitre (TCID_50_/mL). The antiviral activity was assayed using the cytopathic effect inhibition assay, which was expressed as EC_50_. The effect of extract on uninfected Vero cells was given by cytotoxic concentration 50% (CC_50_; *μ*g/mL). The MMML together with several species from the Indonesian Loranthaceae, which was collected from Padang in the State of Andalas, Indonesia, was reported to exert moderate anti-HSV-1 activity with significant activity against Poliovirus. The MMML exhibited CC_50_ against HSV-1 and Poliovirus with value greater than 1000 *μ*g/mL (>1000 *μ*g/mL) or equal to 1000 *μ*g/mL, respectively. In terms of EC_50_, the value recorded for treatment of extract against HSV-1 virus at 20 TCID_50_ and 200 TCID_50_ was 192 and 706 *μ*g/mL, while, for treatment against Poliovirus, the EC_50_ value obtained at 20 TCID_50_ and 200 TCID_50_ was 111 and 225 *μ*g/mL, respectively. 

Nazlina et al. [55] have studied the antiviral activity of MMML. The extract earlier subjected to the TLC assays resulted in the isolation and identification of rutin, quercitrin, and quercetin. The antiviral activity was screened against HSV-1 and vaccine strain of measles (Schwarz) wherein three methods of treatment to detect antiviral activity in each of the fraction were used (see Table 4). For the antiviral tests, the extract was diluted at 1.0 LC_50_, 0.1 LC_50_, and 0.01 LC_50_. The MMML exerted antiviral activity with different modes of action against HSV-1 or measles viruses. The extract was effective in inhibiting cell death by 0.01 LC_50_ in HSV-1-inoculated cells using treatment mode ((C + V) + E) suggesting that virus-inoculated cells were able to overcome viral infection when treated with the extract. Cells treated with simultaneous addition of measles virus and the extract at 0.1 and 1.0 LC_50_ were found to survive from viral infection. The effect of MMML is probably due to the quercetin content that can inhibit reverse transcriptase which is the early part of the measles' replication process. Another possibility is that the MMML is capable of acting directly on viral particle such as modification of free viruses surfaces that inhibits viral attachment to host cells. This belief was supported by the fact that MMML was not capable of inhibiting virus-inoculated cells in treatment mode I. However, the most probable mode of infection can only be further confirmed by transcriptomic or proteomic studies. The extract was found not to have prophylactic effect on both test viruses as demonstrated in treatment mode ((C + E) + V). As for control, guanidine hydrochloride is seen in this study to inhibit viral capability of infecting host cells when added simultaneously, which is believed to happen via blocking of the initiation step of viral RNA synthesis.

### 4.4. Antiparasitic Activity

Alen et al. [67] have screened 65 methanolic extracts of Sumatran rain forest plants, including *M. malabathricum*, for their nematocidal activity against *Bursaphelenchus xylophilus* using the *in vivo* cotton ball- (bl-) fungal mat assay. The authors reported that the MMML, together with that of *Allamanda cathartica, Ervatamia corymbosa, Hoya diversifolia, Bischofia javanica, Derris malaccensis, Ophiorrhiza konsteleary*, and *Brucea sumatrana*, exhibited strong nematocidal activity with the recorded minimum effective dose (MED) of approximately 5 mg/bl. However, the extracts of *Bischofia javanica, Knema hookeriana* and *Areca catechu* were considered to be the most effective nematocidal agents with the recorded MED of approximately 0.7 mg/bl.

### 4.5. Antioxidant Activity

Susanti et al. [22] have also studied the antioxidant activity of the ethanolic solution of the crude EAMMFw and MMMFw, as well as naringenin, kaempferol and kaempferol-3*-O-*D-glucoside isolated from the EAMMFw and kaempferol-3*-O-*(2′′,6′′-di*-O-p-trans*-coumaroyl)-**β**-glucopyranoside and kaempferol-3*-O-*D-glucoside isolated from the MMMFw. The antioxidant assay was carried out by the DPPH radical scavenging electron spin resonance (ESR) spectroscopic method with vitamin E and vitamin C used as standard positive control. The ethanolic solution of the test sample 100 *μ*l (1 mg/mL) was added to 100 *μ*l of DPPH (39.43 M) in ethanol solution and subjected to the assay. At the concentration of 7.8 *μ*g/mL, the MMMFw exerted a stronger free radical scavenger activity than the EAMMFw with the percentage of inhibition recorded at 59.3 ± 1.4% and 53.2 ± 1.3%. Naringenin, kaempferol, kaempferol-3*-O-*D-glucoside, and kaempferol-3*-O-*(2′′,6:-di*-O-p-trans*-coumaroyl)-**β**-glucopyranoside, at the concentration of 7.8 *μ*g/mL, were found to produce 42.5 ± 0.7%, 38.6 ± 0.09%, 24.6 ± 0.3%, and 31.2 ± 4.5% inhibition in comparison to vitamin E and vitamin C, which produced 50.9 ± 0.07% and 82.2 ± 0.3% inhibition, respectively. The IC_50_ recorded for MMMFw, EAMMFw, naringenin, kaempferol, kaempferol-3*-O-*D-glucoside, kaempferol-3*-O-*(2′′,6′′-di*-O-p-trans*-coumaroyl)-**β**-glucopyranoside were 6.59 ± 0.8 *μ*g/mL, 7.21 ± 0.5 *μ*g/mL, 0.52 ± 0.5 mM, 81.5 ± 0.7 *μ*M, 1.07 ± 0.4 mM, 35.8 ± 0.5 *μ*M, respectively. According to Susanti et al. [22] kaempferol-3*-O-*(2′′,6:-di*-O-p-trans*-coumaroyl)-**β**-glucopyranoside was a more active antioxidant than the other compounds and this pronounced radical-scavenging activity is due to the presence of two p-coumaroyl acid groups which are located at the 200 and 600 positions in the glucose ring. Thus, it is proposed that the presence of compound 4 contributed to the higher antioxidant activity of the MMMFw. Meanwhile the authors also suggested that naringenin exhibited a less active antioxidant activity when compared to kaempferol or kaempferol-3*-O-*D-glucoside due to the lack of an unsaturated heterocyclic ring (C-ring), which allows electron delocalization across the molecule for stabilization of the aryloxyl radical, as well as the lack of a 3-OH group.

The antioxidant potential of HMML, EAMML, and MMML of white petals *M. malabathricum*, collected from Johor, Malaysia, together with the isolated compounds (e.g., *α*-amyrin, patriscabatrine and auranamide, quercetin, quercitrin, and kaempferol-3*-O-*(2′′,6′′-di*-O-p-trans*-coumaroyl)glucoside), was determined using the ferric thiocyanate (FTC) and 2,2-diphenyl-1-picrylhydrazyl (DPPH) (UV and ESR spectroscopic) methods [56]. In the former assay, the final concentration of test solution was 0.02% w/v, while, in the latter assay, the concentration of test solutions used were 500, 250, 125, 62.5, 31.3 and 7.8 *μ*g/mL. From the data obtained, kaempferol-3*-O-*(2′′,6′′-di*-O-p-trans*-coumaroyl) glucoside, kaempferol-3*-O-β*-D-glucose, kaempferol, hyperin, quercetin, and quercitrin showed strong antioxidative activities than vitamin E with inhibition of more than 90% in the FTC method. Quercetin, quercitrin, and kaempferol-3*-O-*(2′′,6′′-di*-O-p-trans*-coumaroyl)glucoside) produced the respective percentage of linolenic acid peroxidation of 96.1%, 94.1%, and 92.2%, which is greater than vitamin E (78.3%). In the DPPH assay, quercetin was found to be the most active free radical scavenger in DPPH-UV and ESR method with IC_50_ of 0.69 and 0.65 *μ*M, respectively. The IC_50_ value of quercetin in the DPPH-UV method was greater than that of the positive control, vitamin E (17.1 ± 2.5 mM) and vitamin C (8.3 ± 1.2 *μ*M) and the other flavonoids, namely, quercitrin (74.1 ± 0.4 *μ*M) and kaempferol-3*-O-*(2′′,6′′-di*-O-p-trans*-coumaroyl)glucoside) (308.1 ± 1.7 *μ*M). The percent inhibition of quercetin, quercitrin, kaempferol-3*-O-*(2′′,6′′-di*-O-p-trans*-coumaroyl)glucoside), vitamin E, and vitamin C at concentration 7.8 *μ*g/mL was 57.60%, 15.4%, 7.5%, 7.4%, and 30.8%, respectively.

In addition, Faravani [58] also reported the antioxidant activity of crude MMMR and methanol extracts of *M. malabathricum* shoots (MMMSt) investigated using the DPPH assay. The MMMSt exhibited their radical scavenging activity as indicated by their ability to reduce the stable free-radical DPPH to the yellow-colored diphenylpicrylhydrazine with an IC_50_ recorded at approximately 141.9 *μ*g/mL and 154.5 *μ*g/mL, respectively. However, the extracts antioxidant activity was considered to be lowered than that of the positive control, ascorbic acid, which produced an IC_50_ of approximately 28.6 *μ*g/mL.

### 4.6. Cytotoxic Activity

An attempt to determine the cytotoxic activity of MMML together with nine methanolic extracts from seven Indonesian medicinal plants was also made by Lohézic-Le Dévéhat et al. [32]. All plants were collected from Padang in the State of Andalas, Indonesia, and subjected to the cytotoxic study on two murine cancer cell lines (e.g., 3LL (Lewis lung carcinoma cells) and L1210 (leukaemic cells)) and four human cancer lines (e.g., K562 (chronic myeloid leukaemia), U251 (glioblastoma), DU145 (prostatic adenocarcinoma), and MCF-7 (mammary carcinoma)). The MMML exhibited cytotoxic activity against 3LL, L1210, K562, DU145, MCF-7, and U251 at the IC_50_ values of 19, 21, 67, 113, >400, and 30 *μ*g/mL. Interestingly, MMML exhibited cytotoxic activity with IC_50_ value of <25 *μ*g/mL against both murine cell lines while the cytotoxicity activity against all human cancer cell lines was observed at an IC_50_ value that was >25 *μ*g/mL.

Susanti et al. [22] have investigated cytotoxic activity of the crude EAMMFw and MMMFw and several compounds isolated from the respective crude extract (e.g., naringenin, kaempferol and kaempferol-3*-O-*D-glucoside, kaempferol-3*-O-*(2′′,6′′-di*-O-p-trans*-coumaroyl)-**β**-glucopyranoside, and kaempferol-3*-O-*D-glucoside) against a MCF-7 cell line using the 3-(4,5-dimethylthiazol-2-yl)-2,5-diphenyltetrazolium bromide (MTT) assay. DMSO (0.1%) and tamoxifen were used as negative and positive controls, respectively. The 500 *μ*g/mL EAMMFw caused a change in the cell morphology of MCF-7 cells line, while MMMFw, at the same concentration, did not display any activity. Naringenin and kaempferol-3*-O-*(2′′,6′′-di*-O-p-trans*-coumaroyl)-**β**-glucopyranoside demonstrated a significant anticancer effect against MCF-7 in a dose-dependent manner with IC_50_ values of 1.30 ± 0.002 *μ*M and 0.28 ± 0.004 *μ*M, respectively. The anticancer activity of those compounds was demonstrated to involve cell proliferation and changes in the cell morphology. Thus, naringenin was suggested to contribute to the anticancer activity of the EAMMFw. The failure of MMMFw to exhibit its cytotoxicity effect suggested that the antagonist effects of the compounds present in the extract play an important role in not affecting the cell proliferation. Interestingly, the IC_50_ value of kaempferol-3*-O-*(2′′,6′′-di*-O-p-trans*-coumaroyl)-**β**-glucopyranoside was lower than that of the positive control, tamoxifen (the IC_50_ value was 0.76 ± 0.005 *μ*M). Based on the cell morphology, it was proposed that kaempferol-3*-O-*(2′′,6′′-di*-O-p-trans*-coumaroyl)-**β**-glucopyranoside was active against human breast cell cancer by inhibiting cell proliferation. Meanwhile, naringenin inhibited cell proliferation, followed by cell lyses. Kaempferol-3*-O-*(2′′,6′′-di*-O-p-trans*-coumaroyl)-**β**-glucopyranoside has a free hydroxyl group at position 3 and a parahydroxyl group in ring B, which probably increase the activity of this compound.

Nazlina et al. [55] have also studied the cytotoxic activity of MMML against Vero cell line (African green monkey, *Cercopitheus aethiops *kidney cells) and L929 cells (mouse fibroblast) whereby the cytotoxicity test was carried out according to the microculture method in, at least, two independent experiments in triplicates at different concentrations of MMML using doubling dilutions from initial stock concentration of 1000 *μ*g/mL. Cytotoxicity screening towards Vero and L929 cells showed that MMML was not cytotoxic to both cells with LC_50_ values of 750 *μ*g/mL and >1000 *μ*g/mL, respectively. As for gunaindine hydrochloride that was used as positive control, it was found not cytotoxic in Vero cells with the LC_50_ value of 100 *μ*g/mL while the LC_50_ value for L929 cells was not determined.

### 4.7. Anticoagulant Activity

Manicam et al. [68] have reported on the anticoagulant property of the leaves of *M. malabathricum* collected from the area of Serdang in the state of Selangor, Malaysia. The hot- and cold-WMML and MMML were assayed for their anticoagulant property using the blood samples drawn from healthy volunteer donors (*n* = 36) of both genders (18–50 years old) after screening *via *questionnaire for familial history of cardiovascular diseases and other major coagulopathies. The coagulation parameters used to determine the extracts anticoagulant activity were the activated partial thromboplastin time (aPTT), prothrombin time (PT), and thrombin time (TT). These parameters were carried out in a STA Compact coagulation analyzer with the maximum cut-off time recorded by the coagulation analyzer set at 180 s. Plasma samples were spiked with different concentrations of *M. malabathricum *extracts (ranging between 100 and 1000 *μ*g/mL), heparin (used as positive control), or deionized water (used as vehicle control). Based on the preliminary study, the hot-WMML, in the concentration of 1000 *μ*g/mL, significantly prolonged (*P* < 0.05) the three parameters, namely, aPTT, PT, and TT, in plasma when compared to the normal control plasma. The PT and TT measurements were 20.0 ± 1.3 s and 43.2 ± 0.1 s when compared to control group (13.3 ± 0.5 or 20.1 ± 0.2 s), respectively. Interestingly, the hot-WMML did not clot the plasma samples when tested for aPTT, as evidenced by the maximum cut-off time recorded at 180 s. The cold-WMML and MMML also prolonged aPTT in a significant fashion (*P* < 0.05) with 120.0 ± 0.9 s and 108.0 ± 0.7 s, respectively, in comparison to the 38.9 ± 0.5 s of control plasma. Similar to the hot-WMML, the PT of cold-WMML was prolonging significantly (*P* < 0.05) in comparison to the control. On the other hand, these extracts did not affect the TT significantly. Based on the anticoagulant activity demonstrated above, at the concentration of 1000 *μ*g/mL, Manicam et al. [68] selected the hot-WMML for further study on its effect on clot-based assays. At the concentration ranging between 100 to 1000 *μ*g/mL, the hot-WMML caused prolongation of aPTT in a concentration-dependent manner with significant anticoagulant activity recorded at the concentration beyond 400 *μ*g/mL in comparison with vehicle control. Interestingly, hot-WMML prolonged aPTT beyond 300 s at 900 and 1000 *μ*g/mL, which was comparable to that of 5–1000 *μ*g/mL heparin. The cut-off time to measure clotting times was 300 s; beyond which the plasma samples were rendered noncoagulable. The control plasma was found to record an aPTT of 64.3 and 60.7 s for females and males, respectively. On the other hand, the aPTT of normal pooled plasma (NPP) was 41.0 and 40.5 s for females and males, respectively, and was significantly different (*P* < 0.05) from that of the control plasma. In contrast to aPTT, hot-WMML caused no significant changes for PT and TT at the tested concentration range. However, PT was significantly (*P* < 0.001) prolonged at the highest contration of heparin (1000 *μ*g/mL) while TT was significantly (*P* < 0.001) prolonged at the highest concentration of hot-WMML (1000 *μ*g/mL) or at the range of 5–1000 *μ*g/mL. Overall, the PT assay recorded the lowest coagulation inhibitory activity for hot-WMML.

### 4.8. Platelet-Activating Factor Inhibitory Activity

Jantan et al. [69] investigated the anti-platelet-activating factor inhibitory property of 49 methanol extracts of 37 species of Malaysian medicinal plants, including *M. malabathricum *collected from Kepong, Shah Alam, Selangor, Malaysia. However, the MMML, at the concentrations of 200, 100, 50, 20 and 10 *μ*g/mL, produced <10% inhibitory effect against platelet activating factor (PAF). Cedrol, a known PAF receptor antagonist and at the concentration of 18.2 *μ*g/mL, was used as a standard in the bioassay.

Mazura et al. [70], in their quest for natural anti-inflammatory agents, assessed the potential of **α**-amyrin, betulinic acid, quercetin and quercitrin isolated from *M. malabathricum* to inhibit PAF binding to rabbit platelets using ^3^H-PAF as a ligand. At 18.2 *μ*g/mL, all compounds exerted 67.3, 64.3, 57.4, and 45.4%, while cedrol, as positive control, caused 79.6%, respectively. The tested compounds, at the serial concentration dilution range of 18.2–1.8 *μ*g/mL, produced the percentage of inhibition (%) of 17.9–70.4%, 11.8–65.1%, 4.3–58.9%, and 2.5–44.8%, respectively. The results also indicated that quercetin, quercitrin, **α**-amyrin, and betulinic acid showed inhibition of PAF receptor binding to rabbit platelets with IC_50_ values of 33.0, 45.4, 20.0, and 22.2 *μ*M, respectively. The IC_50_ values of these compounds were comparable to that of cedrol (13.1 *μ*M), which is a known PAF receptor antagonist.

### 4.9. Wound Healing Activity

Sunilson et al. [66] reported the wound healing potential of MMML, collected from the State of Selangor, Malaysia, in the form of ointment when examined in two types of wound model in rats: (i) the excision wound model and (ii) the incision wound model. The extract, prepared as 5% ointment, exhibited a wound healing activity that was comparable with the standard drug, nitrofurazone, which was prepared as 0.2% ointment, in terms of wound contracting ability, wound closure time, tensile strength, and regeneration of tissues at the wound site. The time to wound closure of the nitrofurazone- and the extract-treated groups was the same (18.0 ± 2.0 days). In the incision wound studies, the extract ointment and nitrofurazone caused a significant increase in tensile strength of the 10-day-old wound when compared with the control (418.0 ± 13.8 g). The tensile strength of the extract ointment- and the nitrofurazone-ointment-treated groups was almost the same (551.0 ± 16.9 g versus 576.0 ± 12.5 g). Interestingly, the extract ointment enhanced original tissue regeneration of the skin wounds much greater than nitrofurazone with the standard drug exerting more relative fibrosis of skin wounds when compared to the extract ointment. Although fibrosis was relatively less in the extract ointment-treated rats, the original tissue was regenerated much more in the animal wounds. The skin adrenal structures such as the Pilosebaceous glands and sweat glands were better presented in wounds treated with extract ointment compared to nitrofurazone-treated animal wounds.

### 4.10. Antiulcer Activity

Hussain et al. [71] studied the antiulcer activity of the AMML, collected around the University of Malaya campus in Petaling Jaya, Selangor, Malaysia, against ethanol-induced gastric mucosal injuries in rats. The extract, in the dose of 250 and 500 mg/kg, and 20 mg/kg omeprazole, used as positive control, were administered orally followed 1 hour later by the oral administration of the ethanol. Macroscopically, the oral administration of the test solutions was found to significantly (*P* < 0.05) reduce the formation of gastric mucosal injuries in a dose-dependent manner when compared to the group that was pretreated with only distilled water. The ulcer area recorded for groups pretreated with distilled water, 250 and 500 mg/kg extract, and 20 mg/kg omeprazole was 845.00 ± 52.17 mm^2^, 210.00 ± 8.17 mm^2^, 70.00 ± 8.27 mm^2^ and 30.00 ± 5.32 mm^2^, respectively. In terms of percentage of protection, the respective test solution was found to give approximately 75.15%, 91.72%, and 96.45% protection. Microscopically, distilled-water-pretreated rats exhibited severe damage of the gastric mucosa, and induced submucosal edema and leucocytes infiltration while extract- or omeprazole-received rats exerted marked reduction of gastric mucosal damage, reduction of oedema, and less leucocyte infiltration of submucosal layer. There were no significant differences between the cytoprotective abilities of the animals treated with 20 mg/kg omeprazole compared to the animals treated with 500 mg/kg AMML. Overall, the 500 mg/kg AMML was found to provide the best protection to the gastric mucosa in rats against ethanol-induced gastric ulcers.

### 4.11. Antidiarrheal Activity

The antidiarrheal activity of WMML, collected from the State of Selangor, Malaysia, was investigated using four experimental models of diarrhea in mice [61]. In model 1, mice were given test solutions (e.g., 100, 200, and 500 mg/kg of extract or 5 mg/kg loperamide) and fecal materials were collected for 12 h after treatment, dried in an incubator, and weighed. The percentage reduction in the fecal output was determined. In model 2, an overnight fasted male mouse was induced with diarrhea by oral administration of castor oil (0.5 mL/mouse, p.o.) 1 hour after the test solutions administration. The percentage protection from diarrhoeal droppings was calculated. In model 3, overnight fasted mice were subjected to the enteropooling assay method wherein the animals received the test solutions and, 1 hour later, administered orally with a diarrheal agent, 10% aq MgSO_4_ (0.5 mL/mouse). Thirty min later, the animals were killed and the small intestines were collected and weighed to find out the accumulation of intestinal fluid secretion evoked by MgSO_4_. In model 4, the overnight fasted animals were subjected to the gastrointestinal transit test. The animals received test solutions, and, 5 min later, 0.5 mL of 3% charcoal suspended with tragacanth powder was administered orally to each mouse. Thirty min later, all the mice were killed by cervical dislocation and the distance travelled by the charcoal plug from pylorus to caecum was determined and expressed as a percentage of the total length of the small intestine. From the data obtained in model 1, 100, 200, and 500 mg/kg WMML significantly (*P* < 0.05) reduced the dried fecal output of the mice (dried fecal output per 100 g of mice) by 0.364 ± 0.012 (30.13% reduction), 0.314 ± 0.046 (39.73% reduction), and 0.296 ± 0.023 (43.19% reduction), respectively, while the reduction in the fecal output by loperamide (5 mg/kg) was noted to be 0.222 ± 0.015 (57.39% reduction) when compared to the control group (0.521±0.083). In model 2, the WMML, at its respective dose, significantly (*P* < 0.05) protected the mice against castor-oil-induced diarrheal droppings by 60, 80 and 80%, while loperamide produced the 100% protection. In model 3, the 100, 200, and 500 mg/kg WMML significantly (*P* < 0.05) and dose-dependently reduced the intestinal fluid secretion induced by MgSO_4_, with the weight of the small intestine per 100 g of mice recorded at 8.413 ± 0.431, 7.620 ± 0.469, and 7.314 ± 0.261 when compared to the control group (9.362 ± 0.518). Interestingly, 500 mg/kg WMML caused reduction in the intestinal fluid secretion that was almost comparable with that of 5 mg/kg loperamide (6.416 ± 0.514). In model 4, the 100, 200 and 500 mg/kg WMML significantly (*P* < 0.05) inhibited the small intestinal motility of the charcoal marker in mice in a dose-dependent manner by 8.10%, 25.15%, and 32.97% inhibition in comparison to 5 mg/kg loperamide (57.42% inhibition). The respective distance travelled by charcoal marker for each dose of extract presented as percentage of total length of small intestine was recorded at 63.63 ± 3.71, 51.82 ± 4.11 and 46.41 ± 3.25 when compared to the control group (69.24 ± 5.03) or 5 mg/kg loperamide (29.48 ± 2.69).

### 4.12. Antivenom Activity

Uawonggul et al. [72] reported the antivenom profile of the aqueous extracts of 64 plant species, including *M. malabathricum*, collected from northern and northeastern parts of Thailand and have been listed as animal- or insect-bite antidotes in old Thai drug recipes. The extracts, at the concentration of 0.406 and 0.706 mg/mL, were screened for their activity against fibroblast cell lysis after *Heterometrus laoticus *scorpion venom treatment. *H. laoticus *scorpions were captured from suburban areas of Khon Kaen City, Khon Kaen Province, Thailand. Most of the plants, at the concetration of 0.706 mg/mL, were found to give more than 40% efficiency following cell treatment with venom preincubated with the respective extract with *Andrographis paniculata *Nees (Acanthaceae) and *Barringtonia acutangula *(L.) Gaertn. (Lecythidaceae) given more than 50% efficiency, indicating that they had a tendency to be scorpion venom antidotes. However, AMML only caused 39.86% efficiency at 0.706 mg/mL and 34.41% efficiency at 0.406 mg/mL. The percentage of viable cells after 30 min treatment with 0.706 and 0.406 mg/mL AMML preincubated with 0.2 *μ*g/mL *H. laoticus *venom were 14.84 ± 1.03 and 14.11 ± 1.03 in comparison to their respective mock control, which is 37.23 ± 0.34 and 41.00 ± 0.52, respectively.

### 4.13. Anti-Inflammatory Activity

Zakaria et al. [30] reported on the anti-inflammatory activity of AMML, collected from Shah Alam, Selangor, Malaysia. The anti-inflammatory activity was determined using only the carrageenan-induced paw edema wherein the extract, at the concentration of 10%, 50%, and 100%, was administered via subcutaneous route and measurement of paw thickness was carried out for 8 hours following the extract administration with 1-hour interval. The concentration of AMML used was equivalent to the doses of 4.87, 24.35, and 48.7 mg/kg, respectively. The AMML was found to show significant (*P* < 0.05) anti-inflammatory activity in a concentration-independent manner wherein the activity was seen only at the 50% and 100% concentrations. In terms of the onset of action, the AMML exhibited the anti-inflammatory activity 1 hour after its subcutaneous administration and this activity was seen until the end of experiments. Interestingly, 100 mg/kg acetylsalicylic acid (ASA), used as positive control, exerted similar strength of anti-inflammatory when compared to the extract as indicated by the statistically insignificant data at the respective time interval.

Susanti et al. [56] investigated the anti-inflammatory activity of pure compounds obtained from HMML, EAMML and MMML using the 12*-O-*tetradecanoylphorbol-13-acetate- (TPA-) induced mouse ear oedema assay. The pure compound (20 *μ*L) was applied topically to the inner surface of the right ear of the mice with the left ear receiving only vehicle acting as negative control group. The pure compounds (0.5 mg/ear) and the standard drug indomethacin (0.5 mg/ear) were applied topically, simultaneous with TPA. From the results obtained, 0.5 mg/mL kaempferol-3*-O-*(2′′,6′′-di*-O-p-trans*-coumaroyl) glucoside and *α*-amyrin demonstrated the strongest activities in the anti-inflammatory assay with the IC_50_ of approximately 0.11 ± 0.4 and 0.34 ± 1.1 mM/ear, respectively as compared to the 0.5 mg/mL indomethacin (2.10 ± 0.5 mM/ear). However, no data on the anti-inflammatory effect of those crude extracts were given for comparison with their pure compounds.

### 4.14. Antinociceptive Activity

The ethanolic extract of *M. malabathricum* stem barks and leaves (EMMSBL), collected from Serdang, Selangor, Malaysia, was earlier studied its antinociceptive activity using the acetic-acid-induced abdominal constriction and hot plate test in mice [73]. The extract, administered intraperitoneally in the doses of 30, 100 and 300 mg/kg, was found to exert potential (*P* < 0.05) antinociceptive activity in a dose-dependent manner in the former test with the percentage of nocicptive inhibition recorded at 23.0%, 50.0%, and 84.4%, respectively. The ED_50_ recorded was approximately 100 mg/kg when given intraperitoneally. ASA, at 100 mg/kg dose, produced 79.0% nocicptive inhibition. In the latter test, the extract significantly (*P* < 0.05) increased the response latency period to thermal stimuli in mice also in a dose-dependent manner. The antinociceptive activity of EMMSBL reached its peak approximately 60 min after the extract administration. Morphine, at 5 mg/kg dose, significantly (*P* < 0.05) prolonged the response latency period with maximum effect obtained approximately 1 h after treatment. In an attempt to determine the role of opioid receptors in mediating the EMMSBL antinociceptive activity, 5 mg/kg naloxone, a nonselective opioid antagonist given via intraperitoneal route 15 min before the extract, was found to inhibit the antinociceptive activity of the extract in both tests. 

Zakaria et al. [30] reported on the antinociceptive activity of the AMML, collected from Shah Alam, Selangor, Malaysia. The antinociceptive activity of subcutaneously administered extract, at the concentration of 10%, 50%, and 100% (which is equal to the dose of 4.87, 24.35, and 48.7 mg/kg, resp.), was determined using the acetic acid-induced abdominal constriction, hot plate and formalin test. The AMML was found to show significant (*P* < 0.05) antinociceptive activity in all the three tests. In the acetic-acid-induced abdominal constriction test, all concentrations of AMML caused significant (*P* < 0.05) reduction in the number of abdominal constriction which occur in a concentration-independent manner. The 10%, 50%, and 100% AMML exerted similar strength in antinociceptive activity when compared together or against 100 mg/kg ASA. In the hot plate test, all concentrations of AMML also exhibited a significant (*P* < 0.05) antinociceptive activity in a concentration-independent manner with only the 50% and 100% concentrations of AMML exerting an antinociceptive activity that lasted until the end of the experiment. In this test, 5 mg/kg morphine was used as a positive control and was more effective in increasing the latency to feel thermal-induced pain when compared to the extract. In the formalin test, the AMML at all concentrations used exerted antinociceptive activity in both the early and late phases of the test. The concentration-dependent activity was observed only in the late phase of the test.

### 4.15. Antipyretic Activity

Zakaria et al. [30] also reported on the antipyretic activity of AMML, collected from Shah Alam, Selangor, Malaysia. The antipyretic activity of subcutaneously administered AMML, at the concentration of 10%, 50% and 100%, was determined using the Brewer's Yeast- (BY-) induced pyrexia test. The ability of *M. malabathricum *extract to reduce temperature of pyrexia induced rats was determined for 8 hours with 1-hour interval. The AMML was found to show significant (*P* < 0.05) antipyretic activity at all concentrations tested for the first 6 hours after BY administration. A rapid decrease in the antipyretic activity of the 100% concentration of AMML 3 h after BY administration when compared with the other concentrations of AMML, with completely diminished activity observed for the last 2 h of the experiment. The 100 mg/kg ASA demonstrated antipyretic activity only for the first 4 hours before the activity was gradually lost until the end of experiment.

## 5. Discussion and Conclusion

According to Mitchell and Ahmad [74], the wealth of a country resides to a large extent in its plant inheritance, regardless of whether the plants are endemic, naturalized, or recent introductions. Out of a total of more than 4000 species of Melastomataceae plants in the world, *M. malabathricum* has been one of the 22 species found in the Southeast Asian region alone [1] and one of the 12 species found in Malaysia. Considered as native to tropical and temperate Asia and the Pacific Islands [4], this commonly found small shrub has gained herbal status in the Malay folklore belief as well as the Indian, Chinese, and Indonesian folk medicines. Despite claims that this plant was one of the important herbs within the traditional Malay, Indian, and Chinese medicine, no proper documentation could be found to support them. For example, no documentation that provides proofs for the claims of the importance of *M. malabthricum *in traditional Chinese medicine could be found when the authors carried out thorough search via university or public libraries, as well as the internet. Various parts of this shrub have been claimed to be used in the treatment of various types of ailments and diseases (e.g., diarrhoea, dysentery, leucorrhoea, hemorrhoids, cuts and wounds, infection during confinement, toothache, stomachache, flatulence, sore legs, and thrush) with most of them not yet proven via clinical studies [23]. Despite the lack of clinical studies related to this plant, several attempts have been made to elucidate the pharmacological properties of *M. malabathricum* using the standard and scientific *in vitro and in vivo *techniques of biological evaluations and to finally confirm those folklore claims.

Present interests towards the medicinal benefits of herbal medicines have been increasing worldwide as can be seen with increased laboratory investigation into the pharmacological properties of various medicinal plants. Scientists have also been involved in the isolation, identification, and determination of the bioactive ingredients with specific ability to treat various diseases [75]. Various drugs have entered the international market as a result of scientifically in-depth and systematic exploration of ethnopharmacology and traditional medicine. Despite the increase in scientific study towards medicinal plants all over the world, a smaller number of phytochemical entities or drugs have entered the local or international market due to their evidence-based therapeutics [74]. Due to the latter market trend, efforts are needed to ascertain and confirm evidence regarding safety and practices of plant-based medicines. Furthermore, plant-based medicines are erroneously considered safe because they are regarded as naturally occurring and of plant origin. According to Yob et al. [76], the lack of scientific and clinical data has led to poor understanding of the efficacy and safety of the herbal drugs, which in turn contributes to major impediment in the use of plant-based medicinal preparations. The only attempt to establish acute toxicity profile of *M. malabathricum* was performed by Sunilson et al. [61]. The authors reported the nontoxic effect of WMML up to the dosage of 2000 mg/kg when given to mice. This indicates that the WMML is safe for consumption even at the highest dosage (2000 mg/kg) tested and justifies the traditional uses of *M. malabathricum*.

The dosage range used for *in vivo* study was very important to corroborate with the dosages used in traditional medicine and the selection of dosage range should not exceed the maximum tolerated dose (MTD) of 1000 mg/kg/day suggested for *in vivo *studies [77]. Based on MTD, the dosage range used for *in vivo *antiulcer, antidiarrheal, antinociceptive, anti-inflammatory, and antipyretic activities, which is between 4 and 500 mg/kg, was considered acceptable. In the *in vitro* studies, the pharmacological activities shown have to be interpreted cautiously depending on the EC_50_ or IC_50_ value obtained for the respective study [76]. According to Meyer et al. [78] for any compounds/extracts to be considered active, they need to exhibit the respective activity at EC_50_ or IC_50_ value of less than or equal to 30 *μ*g/mL (≤30 *μ*g/mL). Based on this suggestion, the antibacterial, antiviral, antiparasitic, and antivenom activities were observed at EC_50_ or IC_50_ values that are unrealistic and greater than 30 *μ*g/mL, and, thus, should be ignored. Some of the potential activities of *M. malabathricum*, as indicated by EC_50_ or IC_50_ value of ≤30 *μ*g/mL, include antifungal, antioxidant, and cytotoxic activities. The MMML, CMML, and AcMML exhibited antifungal activity against *C. gloeosporioides* with MIC of 20 *μ*g/mL [64]. The MMMFw and EAMMFw exerted antioxidant activity in the DPPH assay at the IC_50_ of 6.59 ± 0.8 *μ*g/mL and 7.21 ± 0.5 *μ*g/mL, respectively [22]. The MMMFw was also reported to show cytotoxic effect against 3LL, L1210, and U251 cell lines at the IC_50_ values of 19, 21, and 30 mg/mL, respectively [32]. Another major flaw in some of the reports cited above was failure of the authors to provide proper comparison with reference drug [76]. The importance and reason for choosing certain routes of administration and *in vitro* rather than *in vivo* assays in some of the studies have been discussed by Yob et al. [76].

In this paper, we intended to briefly summarize the *in vitro* and *in vivo *assays applied in the discovery of possibly new pharmacological agents from *M. malabathricum*. In addition, various literatures pertinent to the pharmacological investigation of *M. malabathricum* were reviewed to gather all information related to the ethnobotanical, phytochemical, and pharmacological properties of *M. malabathricum*. Although various scientific papers were published on pharmacological properties of *M. malabathricum*, detailed and careful analysis revealed that *M. malabathricum* only exhibited promising antiulcer, antidiarrheal, antinociceptive, anti-inflammatory, and antipyretic activities as measured via the various *in vivo *assays and antifungal, antioxidant, and cytotoxic activities as measured by the *in vitro* assays.

Despite the various medicinal uses of *M. malabathricum* as described in the ethnobotanical section, the therapeutics efficacy of this plant has not been fully studied indepth. Even though there are various types of bioactive compounds isolated and identified from *M. malabathricum* as highlighted in the phytochemical section, their contribution towards the plant claimed medicinal uses or demonstrated pharmacological activities were also not fully studied. Thus, the quest for new compounds from *M. malabathricum* with specific pharmacological activity remains unsolved. It is suggested that researches should be increased to isolate, identify, and collect most of the reported new compounds from *M. malabathricum* so that their pharmacological potential could be investigated thoroughly if they were to be developed as candidates for new drug development in the future. In conclusion, it is hoped that this paper will serve as an encouragement for others to further explore the pharmacological potentials of *M. malabathricum *with hope of developing it as a new therapeutic agents as it is considered as one of the important herbs, particularly in the Malay folklore medicine.

## Figures and Tables

**Table 1:** The vernacular name of *M. malabthricum*.

No.	Vernacular names	Language	Country	References
1	Senduduk, Sekeduduk, Kenduduk	Malay	Malaysia	Abdul Majid and Ting [6] Fazlin et al. [7]

2	Kendudu, Pucuk Kenduduk	Riau		Grosvenor et al. [8]
	Harendong	Sunda	Indonesia	Abdul Majid and Ting [6]
	Kluruk, Senggani	Jawa		Fazlin et al. [7]

3	Singapore rhododendron	English	Singapore, Britain	Ling et al. [4]
	Malabar melastome		Britain	Umali-Stuart and Stiuart-Santiago [9]

4	Mang kre, Mang re, Bre, Kadu-da	Thais	Thailand	Fazlin et al. [7]

5	Malatungau, Malatungaw	Ibanag		
	Bubtoi	Sambali	Philippine	Umali-Stuart and Stiuart-Santiago [9]
	Yagomyum	Cebu Bisaya		

6	Ye mu dan	Chinese	China	Zhengyi et al. [10]

7	Builukhampa	Mizoram		Sharma et al. [11]
	Yachubi	Manipur (Meitei tribe)		Khumbongmayum et al. [12]
	Longumpo, Bobuchunmei	Manipur (Naga tribe)		Ringmichon et al. [15]
	Karali	Malkangiri	India	Pattanaik et al. [13]
	Chuthuksuru	Wokha (Loga-Naga tribes)		Jamir et al. [17]
	Kechi-yaying	Arunachal Pradesh (Adi tribes)		Kagyung et al. [16]
	Koroli	Mayurbhanj		Thatoi et al. [14]

8	Koiam-pay-bang	Bandarban (Marma tribe)		Rahmatullah et al. [18]
	Kakkhu	Netrakona (Garo tribe)	Bangladesh	Rahmatullah et al. [19]
	Aksio	Chittaggong (Chakma, Murong, Tonchonga tribes)		Rahmatullah et al. [20]

**Table:** (a)

Plant parts	Medicinal uses	Reference
Leaves	Leaves are chewed up, pounded, and applied as paste on cuts or wounds or finely chopped up and squeezed to apply the juice onto the wound to stop bleeding	Latiff and Zakri [29]; Jaganath and Ng [25]; Zakaria et al. [30]
	Leaves are used to prevent scarring from smallpox, to treat dysentery, diarrhoea, piles, and as a tonic	Sharma et al. [11]
	Young leaves are eaten to treat diarrhea	Jaganath and Ng [25]
	Young premature leaves are consumed raw to cure dysentery	Sajem and Gosai [31]
	Leaves are also useful to treat ulcers, gastric ulcers, scar, pimple, and black spot at skin	Lohézic-Le Dévéhat et al. [32]
	Combination of leaves and roots in powder form is applied to wounds and pox scars to aid the healing process or used to relieve the discomfort of hemorrhoids	Burkill [24]; Fazlin et al. [7]
	Powdered leaves alone is used as astringent for dysentery	Umali-Stuart and Stiuart-Santiago [9]
	Juice of leaves and roots is used as a digestive aid	Umali-Stuart and Stiuart-Santiago [9]
	Combination of leaves and flowers is used in the treatment of cholera, diarrhoea, prolonged fever, dysentery, leucorrhoea, wounds, and skin diseases and for the preparation of gargles	Perry [36]; Burkill [24]; Koay [23]; Sharma et al. [11]
	Combination of leaves and flowers is used as astringent in leukorrhea and chronic diarrhea	Umali-Stuart and Stiuart-Santiago [9]

Shoots	Shoots are ingested to treat puerperal infections, high blood pressure, and diabetes	Burkill [24]; Koay [23]
	Juice of shoots is used as a mouthwash to relieve a toothache or to treat leukorrhea	

Roots	Roots are used as mouthwash to relieve a toothache and to treat epilepsy	Burkill [24]; Jaganath and Ng [25]; Lohézic-Le Dévéhat et al. [32]
	Roots are given to postpartum women to aid healing and womb strengthening	Fazlin et al. [7]; Jaganath and Ng [25]; Zakaria et al. [30]
	Roots are used to alleviate rheumatism, arthritis, and tenderness in the legs	Burkill [24]; Koay [23]
	Decoction of roots is used to treat diarrhea	Lin [33]
	Juice of roots is applied to lessen the soreness due to thrush in children	Burkill [24]; Koay [23]
	Combination of roots and leaves in a form of decoction or roots alone are used to tone up the uterus after childbirth in order to strengthen the womb and accelerate wound healing, reduce excessive menstrual bleeding and cramps, relieve postmenstrual syndrome, stomach ache, and white discharge, and enhance fertility	Koay [23]

Barks	Barks are useful for the treatment of various skin diseases	Jain and De Filipps [34]

Flowers	Flowers are used to treat cancer	Mohandoss and Ravindran [35]
	Flowers are used as a nervous sedative and for hemorrhoidal bleeding	Umali-Stuart and Stiuart-Santiago [9]
	Combination of flowers, seeds, and leaves is used to reduce white vaginal discharge and indigestion	Jaganath and Ng [25]

**Table:** (b)

Communities/tribes	Country	Medicinal uses	Reference
Gayo and Alas	Aceh, Sumatra, Indonesia	The cold infusion of *M. malabthricum *flowers is an optional ingredient added to an oral remedy for anaemia associated with gastrointestinal bleeding and epigastric pain	Elliott and Brimacombe [37]

Talang Mamak	Riau, Sumatra, Indonesia	The ground leaves are applied as a compress to cuts and wounds	Grosvenor et al. [8]

Malay	Machang, Kelantan, Malaysia	The fruit juice is applied on dry lips	Ong and Nordiana [38]
	Gemenceh, Negri Sembilan, Malaysia	The pounded leaves are applied onto wounds to accelerate healing	Ong and Nordiana [39]

Jah Hut	Jerantut, Pahang, Malaysia	The roots are applied as decoction to treat diarrhea	Lin [33]

Lakher and Pawi	Mizoram, India	The decoction of the leaves or its juice is taken orally to treat diarrhoea and dysentery	Sharma et al. [11]

Meitei	Manipur and Mayurbhanj, Orissa, India	The bark and leaves are used for treating skin troubles, leukorrhea, diarrhea, and dysentry	Thatoi et al. [14]

Didayi	Malkangiri, Orissa, India	The leaves are applied externally as paste to treat cuts and wounds	Pattanaik et al. [13]

Sundanese	Bogor, West Java, Indonesia	The leaves is used as topical application or oral ingestion to treat toothache and for postpartum remedy	Roosita et al. [40]

Marmas	Bandarban, Bangladesh	The root juice is used to treat jaundice	Rahmatullah et al. [18]

Garo	Netrakona, Bangaldesh	The leaf juice is used as a diuretic and to treat various urinary problems	Rahmatullah et al. [19]

Murong	Rangamati, Bangladesh	The root juice or water extract of boiled roots are used orally to treat leukorrhea	Rahmatullah et al. [20]

Naga	Manipur, India	The fresh and dry leaves are used to treat cuts and wounds, stomach disorder, and fever	Ringmichon et al. [15]
	Tahiti	The plant is used to treat diarrhea and dysentery with its bark decoction used as gargle	Umali-Stuart and Stiuart-Santiago [9]

**Table:** (a)

Class of compounds	Presence (+) or absence (−)	Plant part	Reference(s)
Flavonoids	+	Leaves	Zakaria et al. [30]; Simanjuntak [57]
		Aerial	Lohézic-Le Dévéhat et al. [32]
	+	Leaves and roots	Faravani [58]

Flavan-3-ols	+	Aerial	Dinda and Saha [47]; Mohandoss and Ravindran [35]

Triterpenes	+	Leaves	Zakaria et al. [30]; Simanjuntak [57]
	+	Leaves and roots	Faravani [58]

Tannins	+	Leaves	Zakaria et al. [30]; Simanjuntak [57]
	+	Aerial	Lohézic-Le Dévéhat et al. [32]; Dinda and Saha [47]; Mohandoss and Ravindran [35]
	+	Leaves and roots	Faravani [58]

Anthocyanins	+	Aerial	Dinda and Saha [47]; Mohandoss and Ravindran [35]

Saponins	+	Leaves	Zakaria et al. [30]; Simanjuntak [57]
	+	Leaves and roots	Faravani [58]

Alkaloids	−	Leaves	Zakaria et al. [30]

Steroids	+	Leaves	Zakaria et al. [30]; Simanjuntak [57]
	+	Leaves and roots	Faravani [58]

Glycosides	+	Leaves	Simanjuntak [57]
	+	Leaves and roots	Faravani [58]

Phenolics	+	Leaves and roots	Faravani [58]

**Table:** (b)

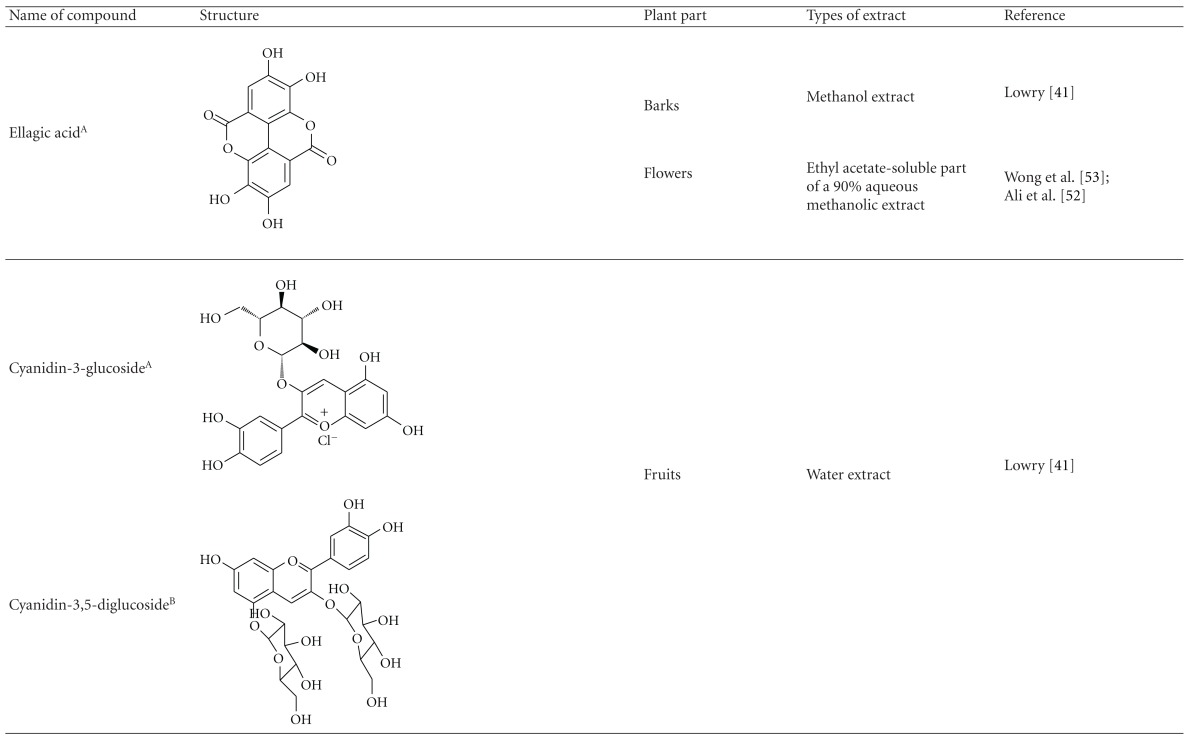 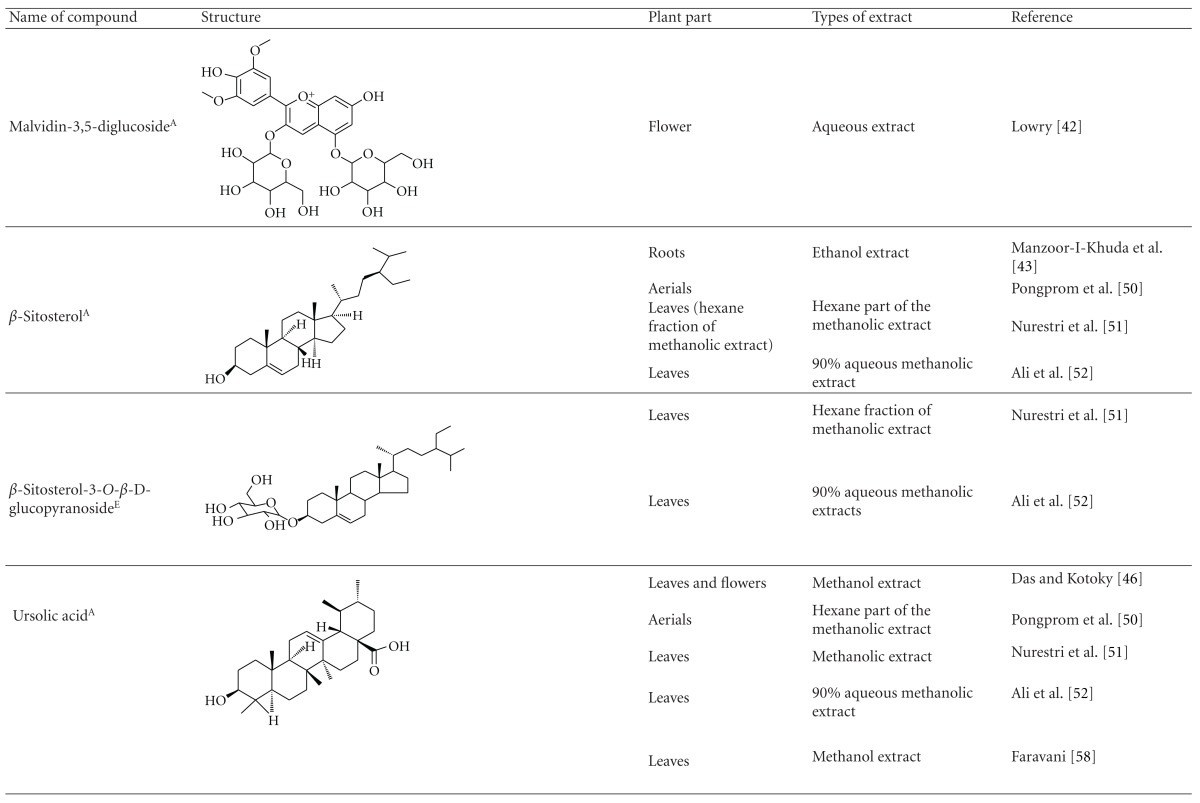 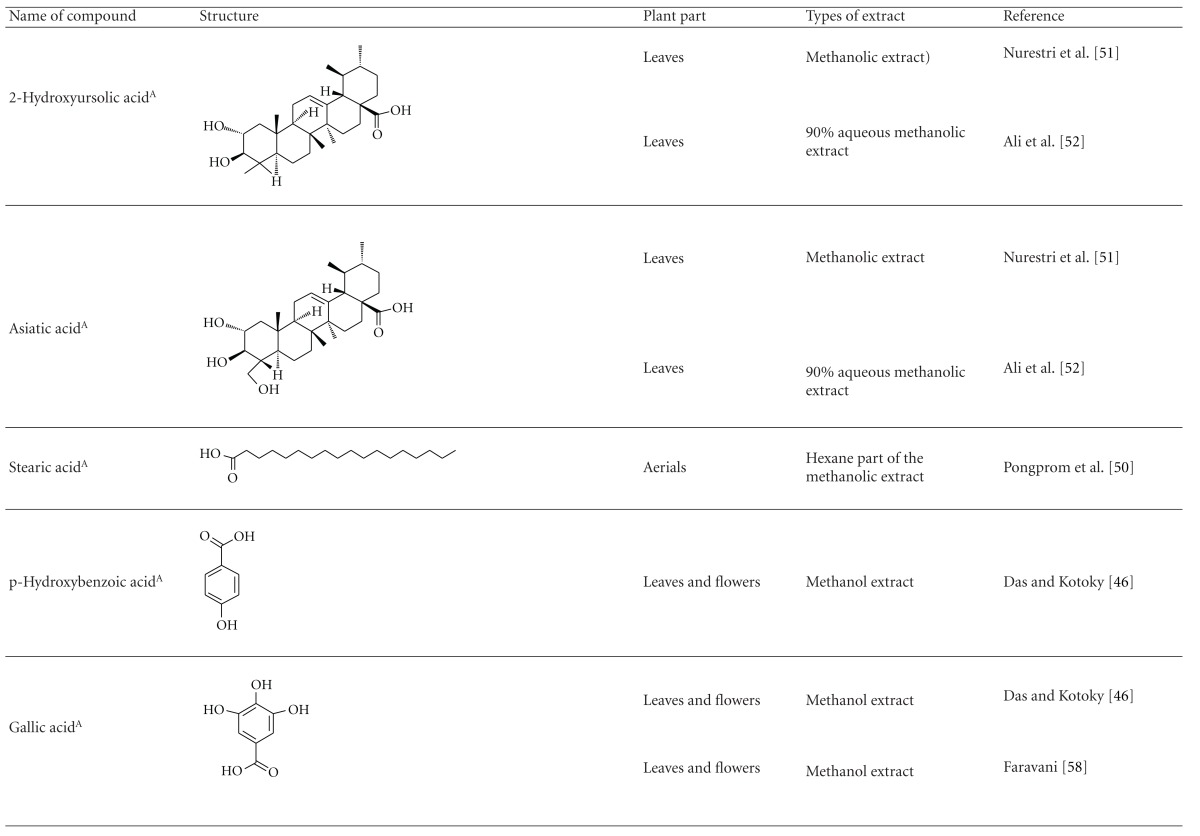 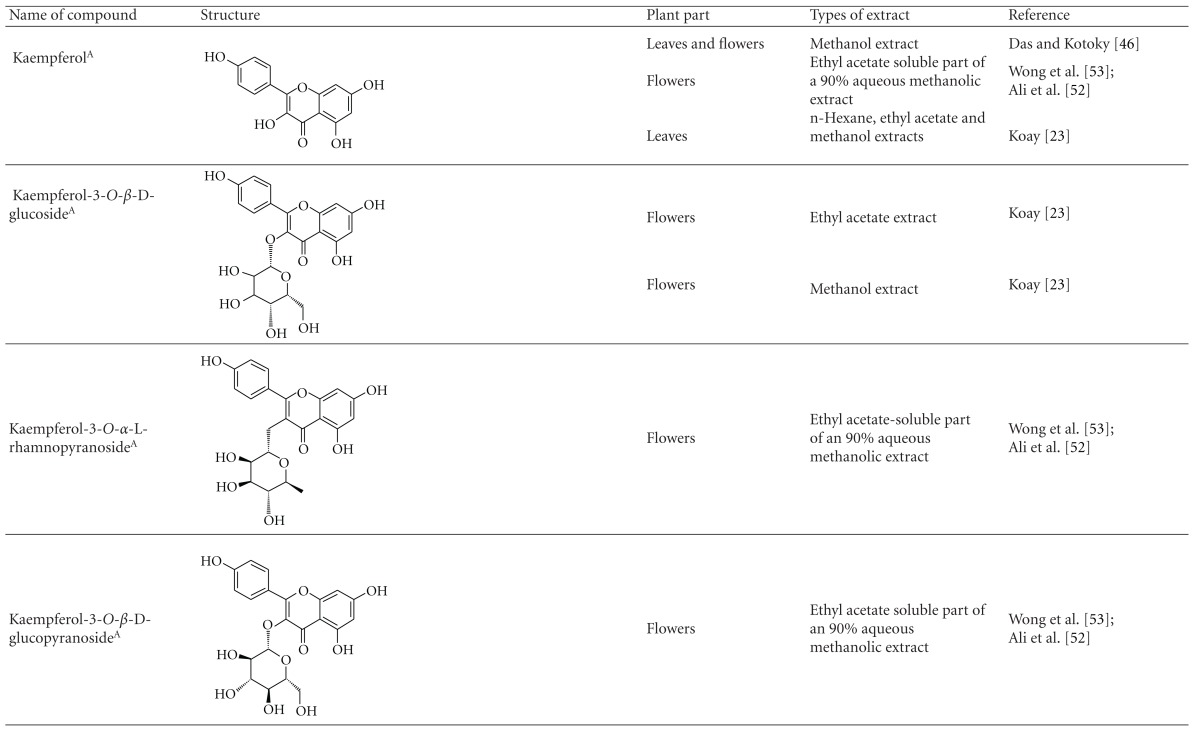 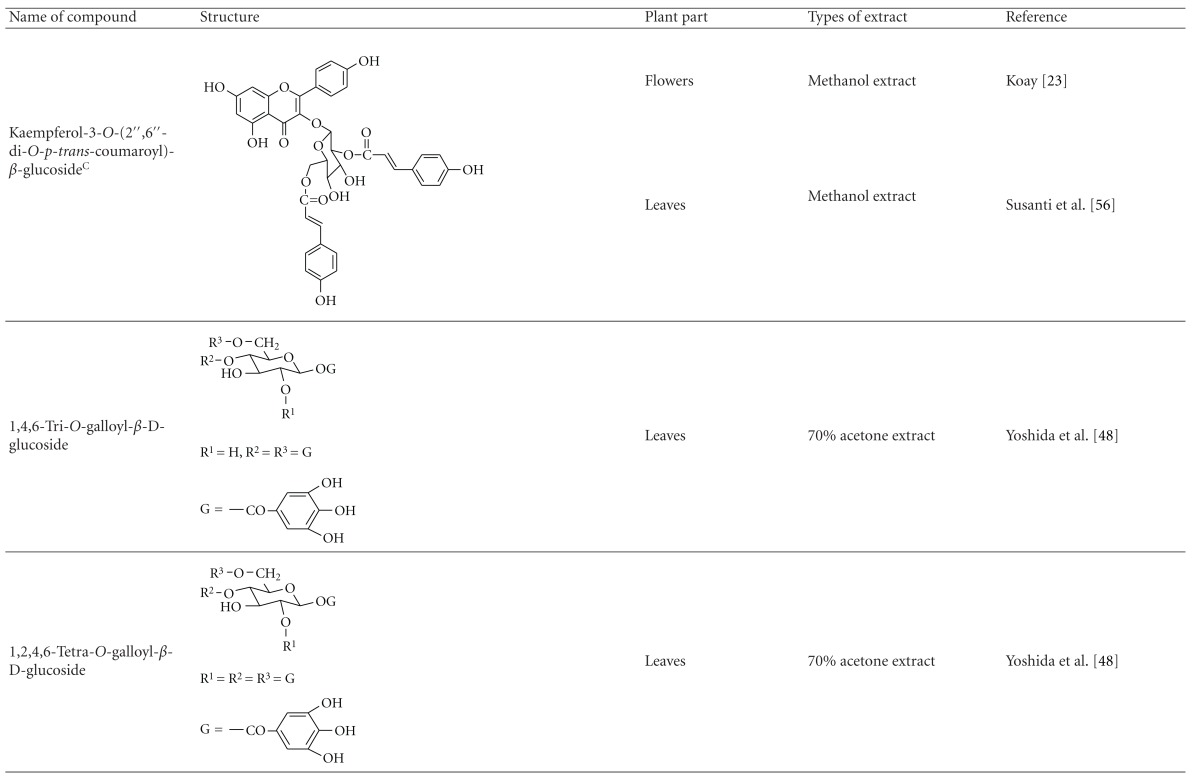 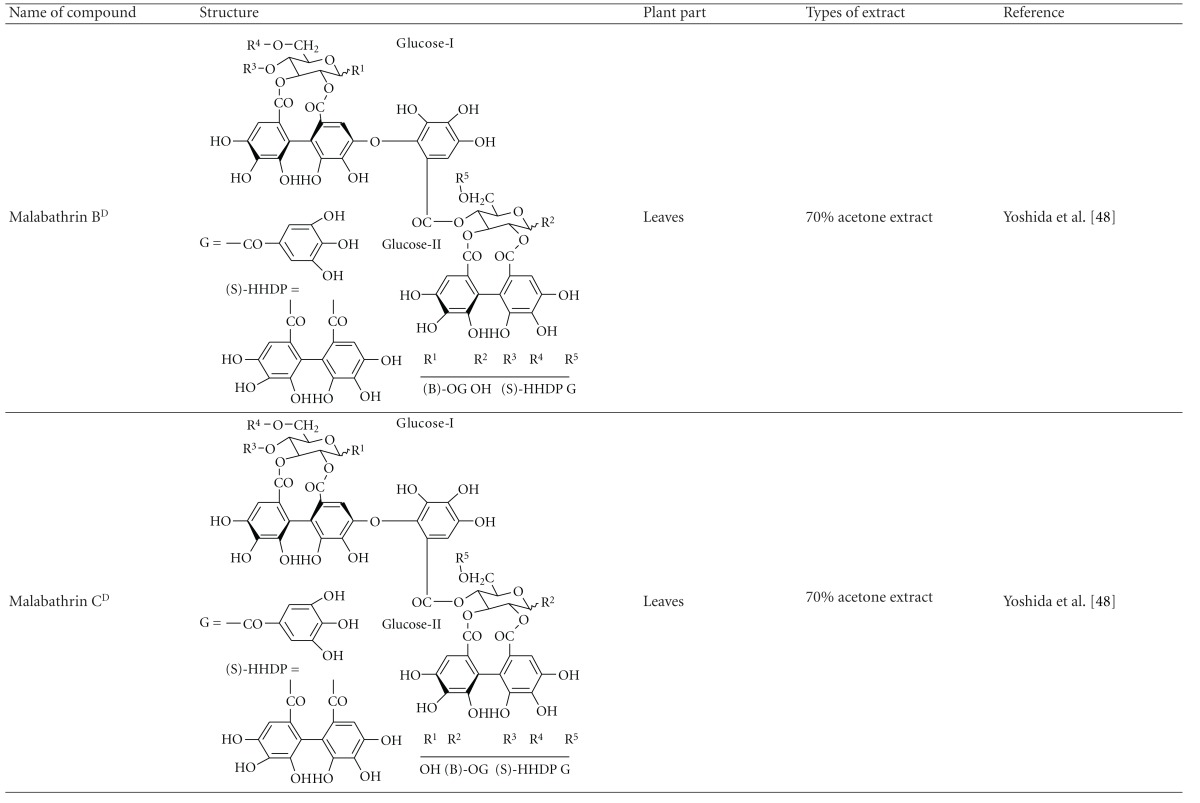 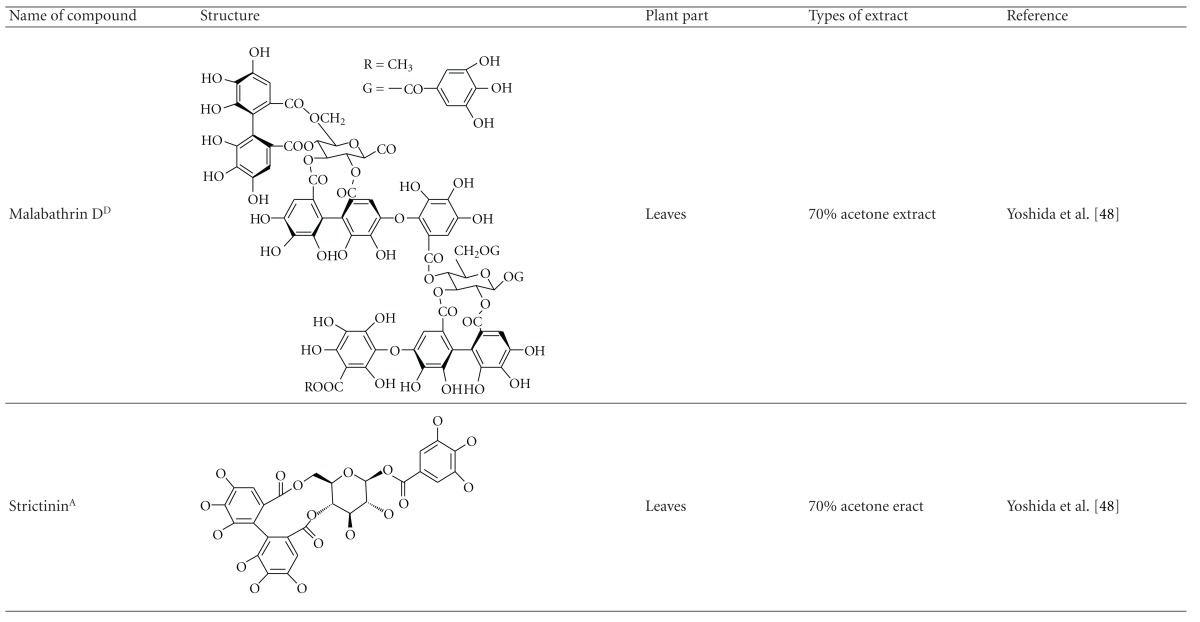 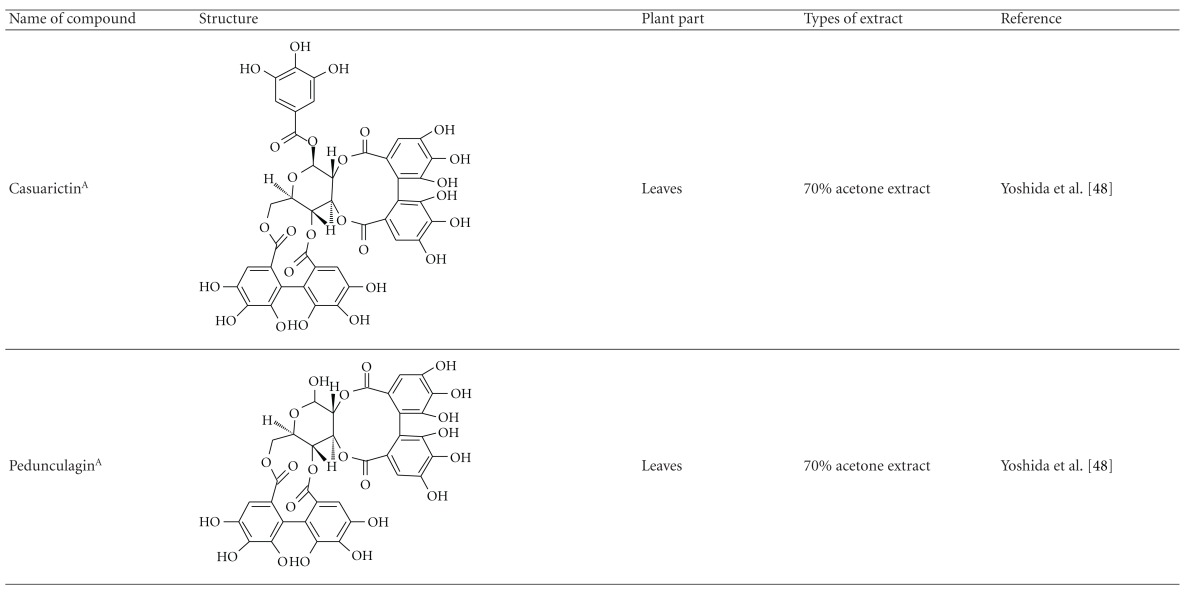 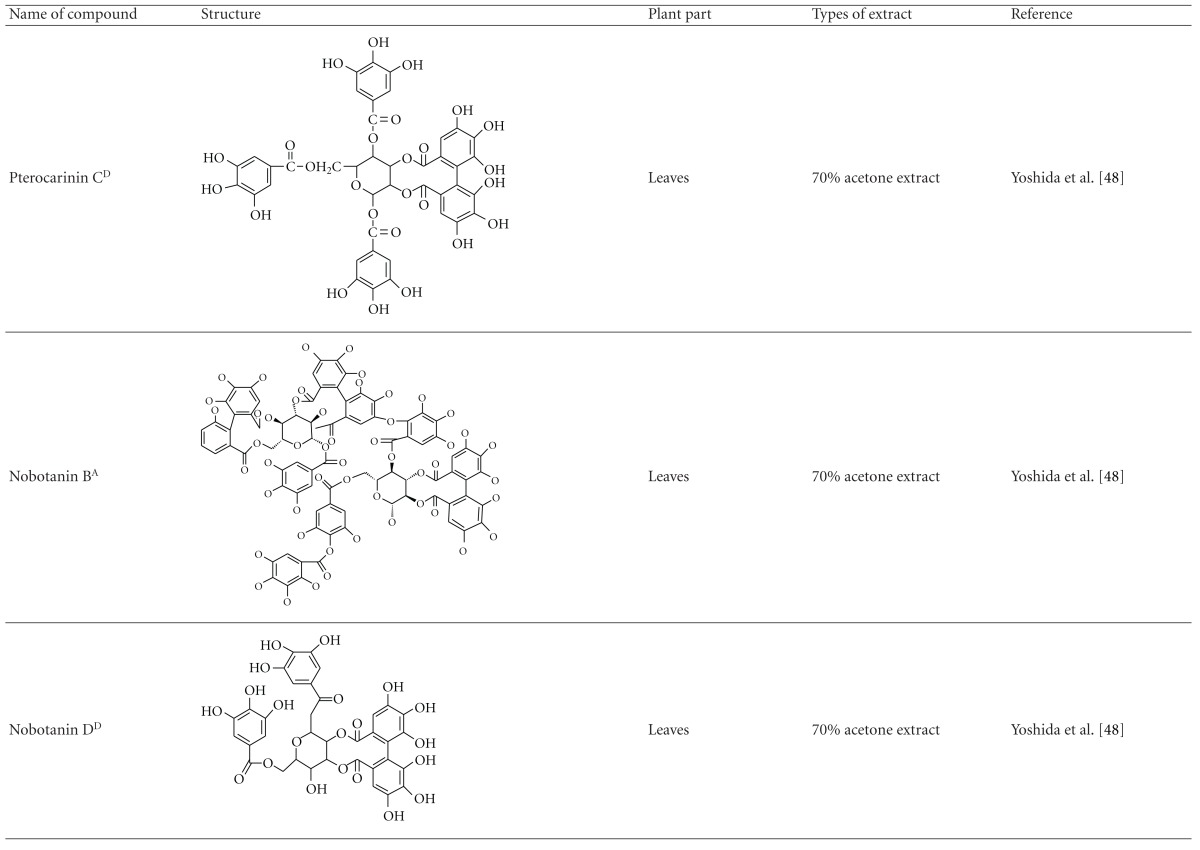 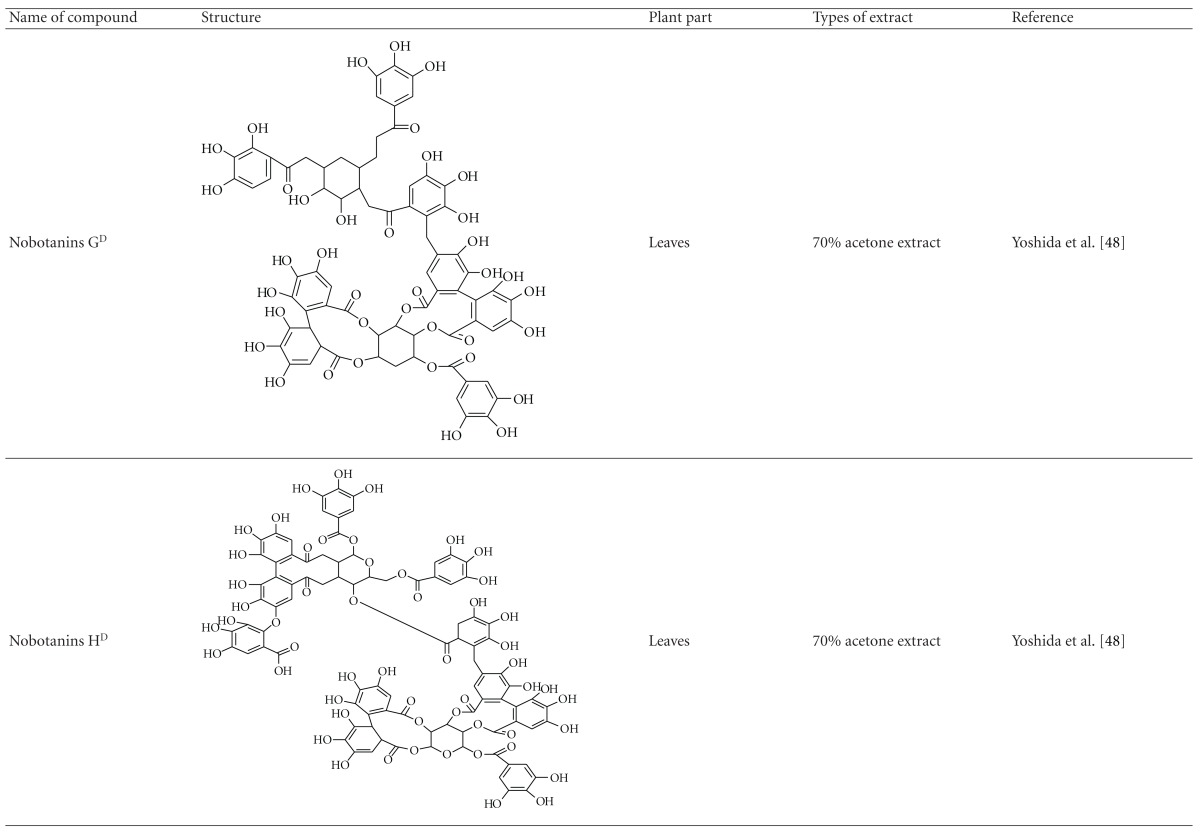 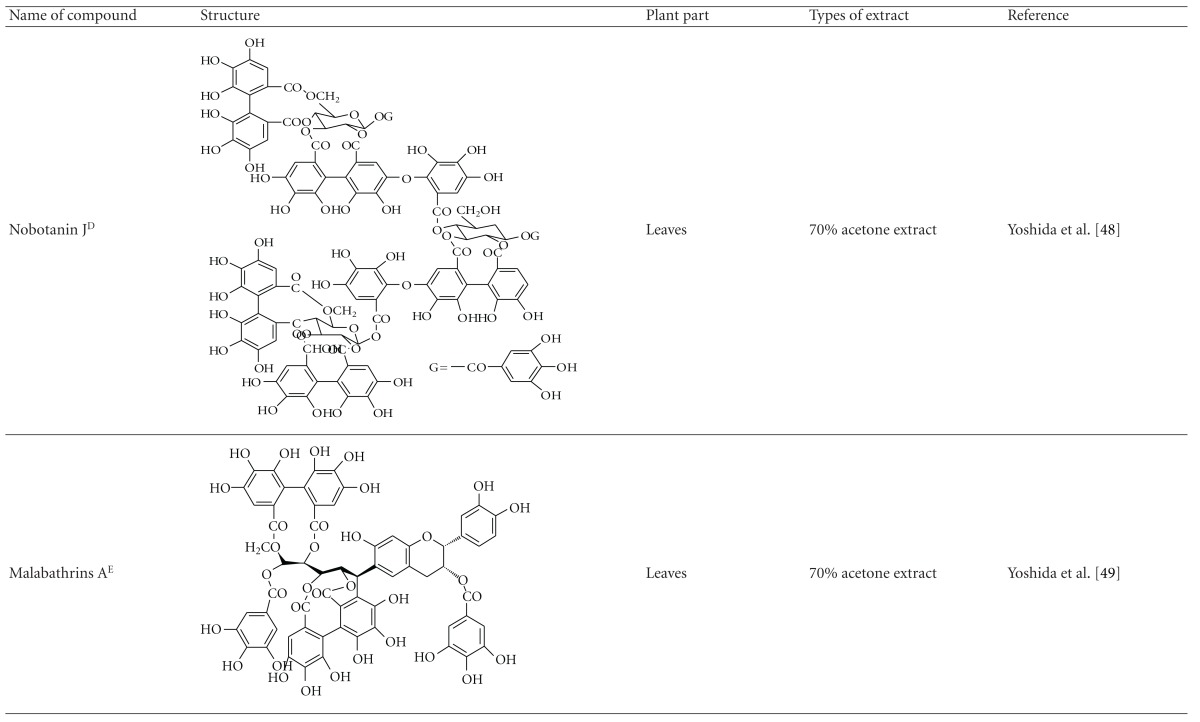 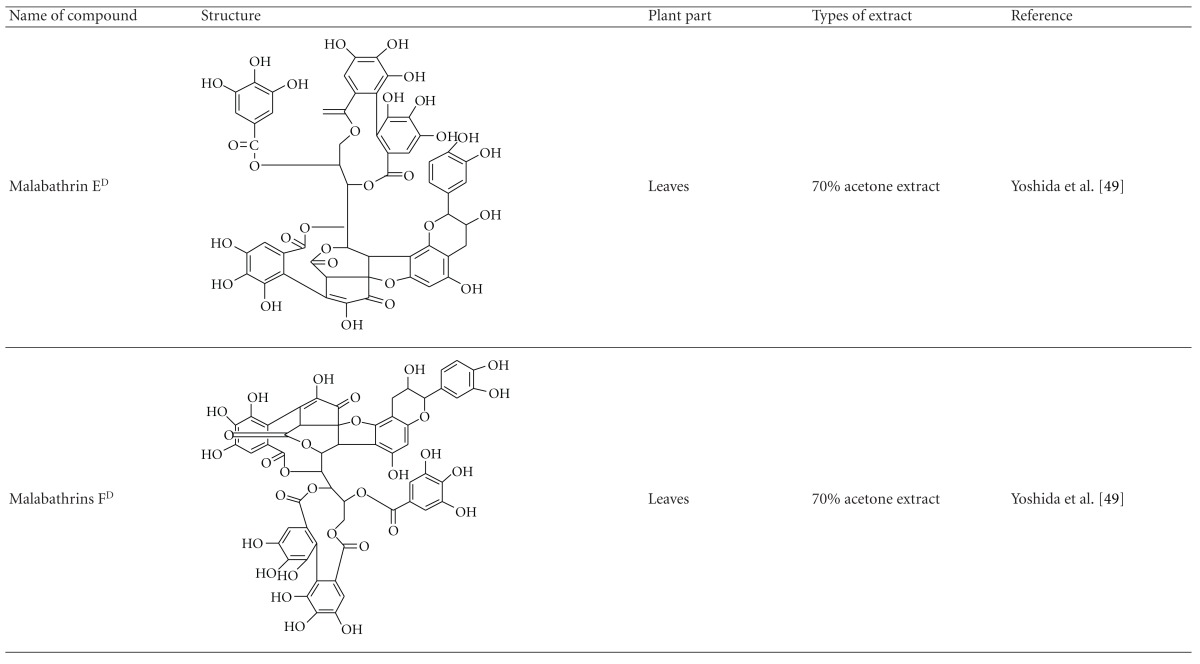 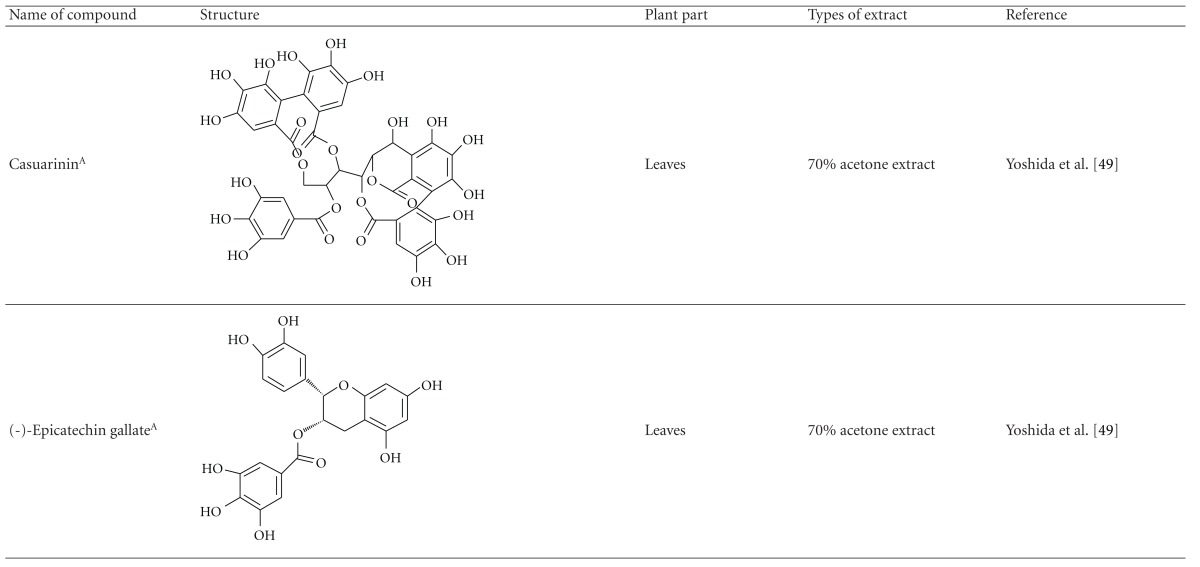 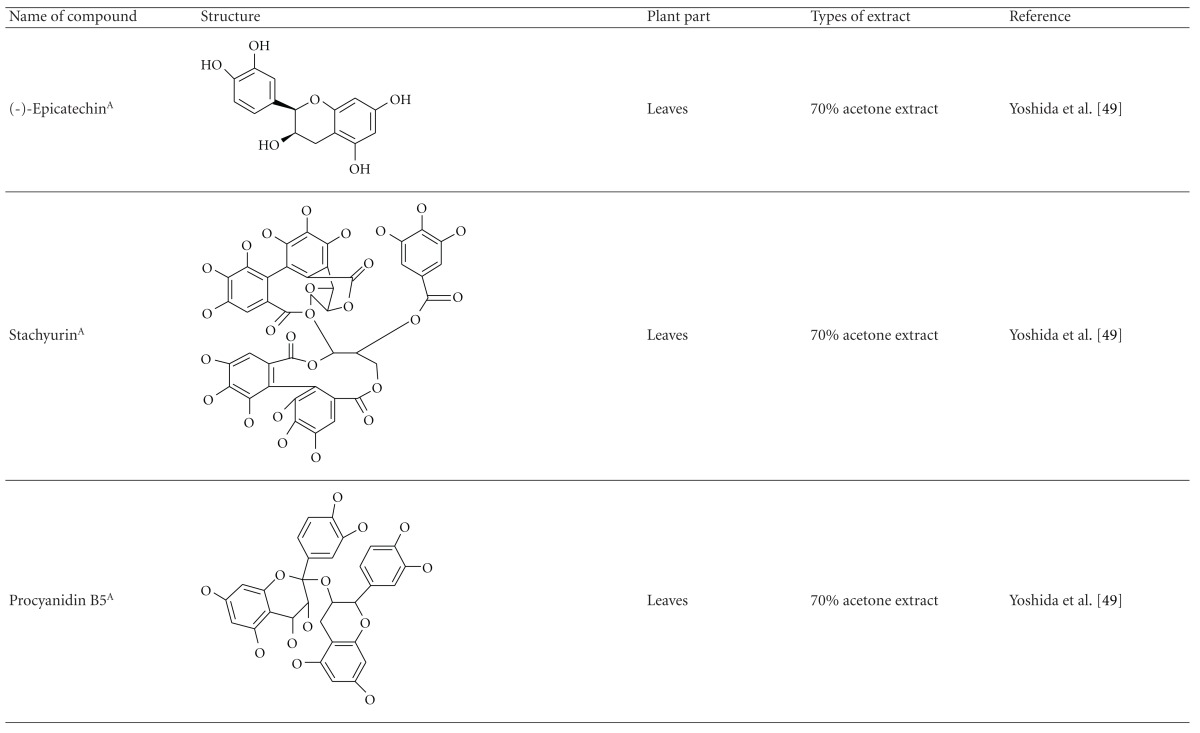 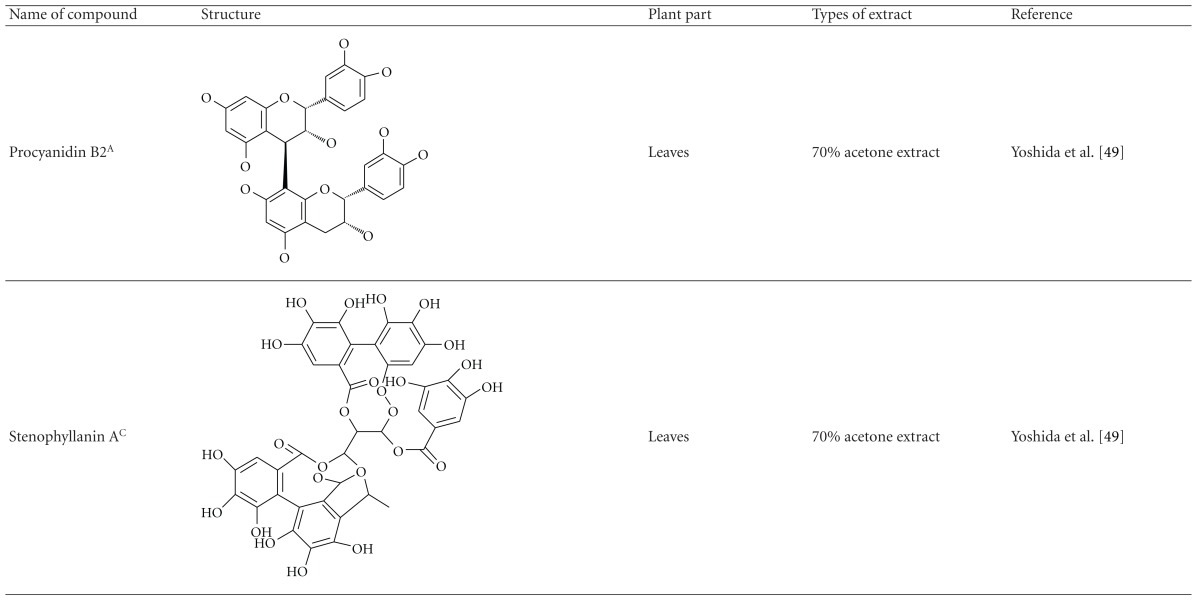 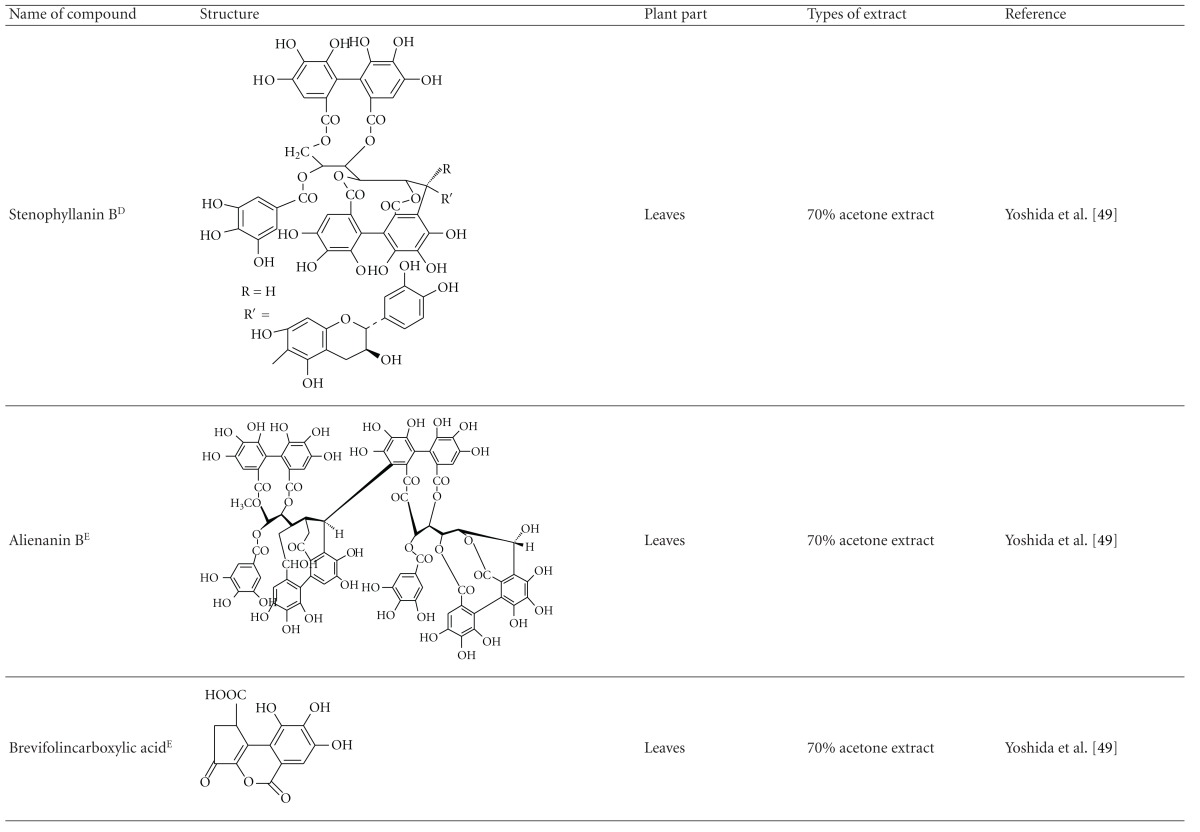 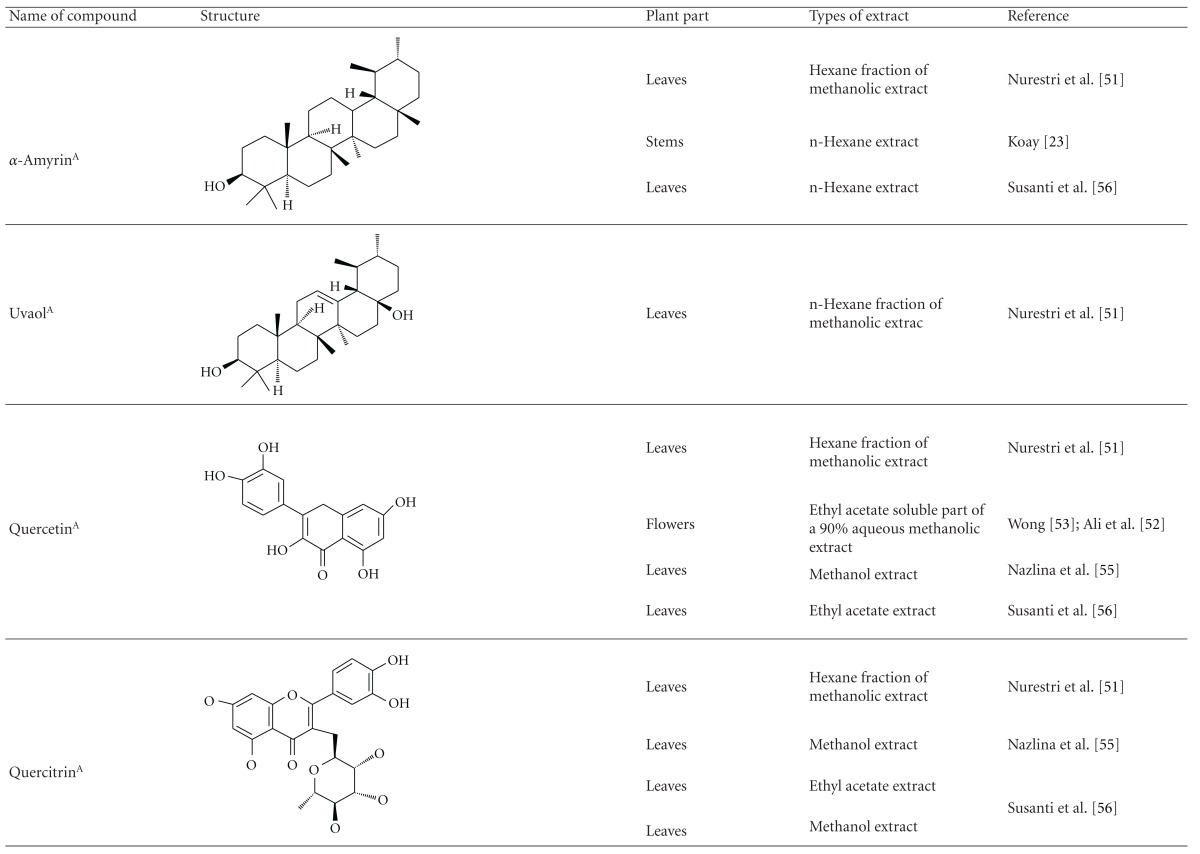 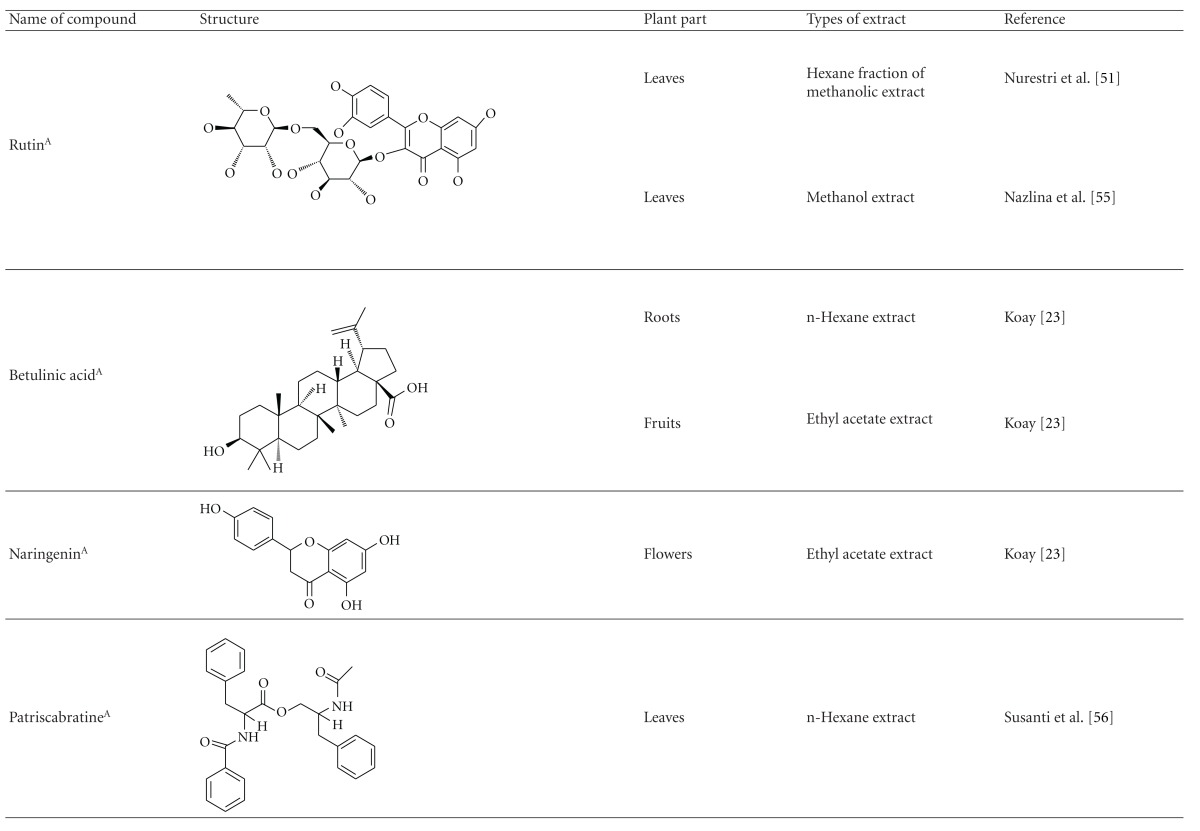 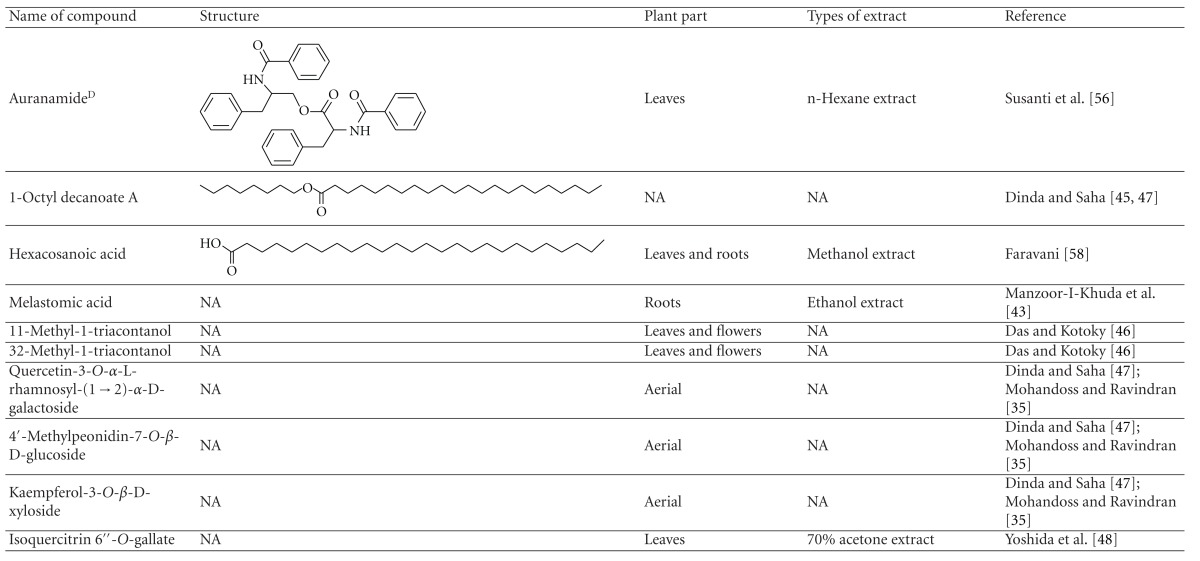 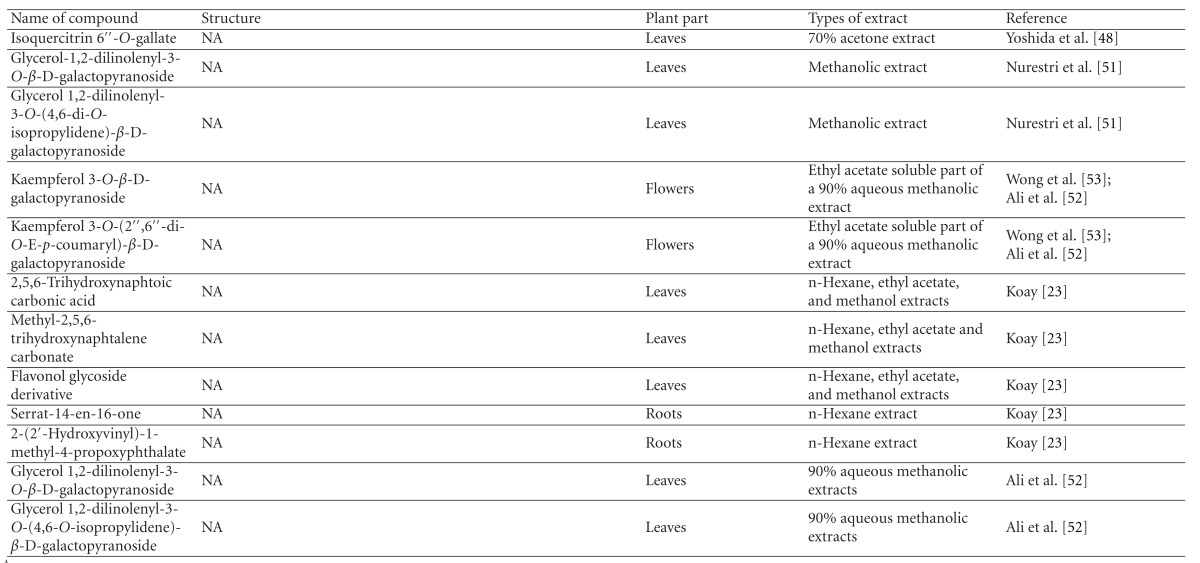

^A^Chemical structure adopted from http://www.ncbi.nlm.nih.gov/.

^B^Chemical structure adopted from http://www.polyphenols.com/.

^C^ Chemical structure adopted from http://www.wikipedia.com/.

^D^Chemical structure adopted from http://www.guidechem.com/.

^E^Chemical structure adopted from the respective reference.

**Table 4:** Pharmacological properties of *M. malabathricum* according to its part.

Pharmacological activity	Pharmacological assay used	Plant part	Types of extract	Dose (mg/kg) or concentration (mg/mL)	Observations	Reference(s)
Acute toxicity	Signs of toxicity were observed for 48 h in orally fed mice	Leaves	Water extract	62.5, 125, 250, 500, 1000, and 2000 mg/kg; given orally	The WMML given to mice up to the dose of 2000 mg/kg showed neither mortality nor any visible clinical signs of general weakness in the animals	Sunilson et al. [61]

Antibacterial	Agar diffusion assay	Combination of leaf, stem, and flower	70% methanol extract	Concentration used was not clearly described 20 *μ*L extract was poured into wells	The AMMMlsf was effective only against *S. aureus*, *S. cerevisiae,* and *F. oxysporum* but not *E. coli*.	Grosvenor et al. [8]
	Disc diffusion assay	Leaves	Methanol extract	Only 1.0 mg/mL extract used	The MMML exhibited poor antimicrobial activity only against *B. subtilis*, but not* B. cereus, E. coli*, *P. aeruginosa*, and* C. albicans*.	Wiart et al. [63]
	Agar cup assay	Different part (leaf, stem, bark, bulb, fruit and root)	Aqueous extract	100 *μ*L of 200 mg/mL extracts was poured into wells	All extracts exerted antimicrobial activity. The AMML exhibited the most effective antimicrobial activity. The AMML was effective against *B. brevis*, *V. cholerae*, *C. krusei*, and *B. subtilis*, while the AMMBk was effective against *S. aureus*, *B. brevis*, and* V. cholera*.	Thatoi et al. [14]
	Agar-disc dilution assay	Leaves	Methanol, chloroform, and acetone extracts	0.01, 0.10, 1.00, and 10.00 *μ*g/mL. 1 mL of each extract was mixed with 19 mL of media and poured into petri dishes. Mycelial discs were inoculated at the center of agar medium	The extracts showed antifungal activity against *C. gloeosporioides*. The MIC value obtained for all extracts was 20.00 *μ*g/mL. The extracts also inhibited the sporulation of tested fungus	Johnny et al. [64]
	Agar well diffusion assay	Leaves	Water, benzene, and acetone extracts	50 *μ*L of 1.0 mg/mL extract per well	All extracts possessed moderate antimicrobial activity against *E. coli *(MDR), *S. aureus *(MDR), *K. pneumoniae, B. cereus, V. cholera*, and *C. albicans*. The MIC value for all extracts ranged between 0.65 and 0.80 mg/mL, while the MBC value was 0.90 mg/mL against *V. cholera*. The MIC value for WMML and AcMML was 0.80 and 0.79 mg/mL, respectively, while the MBC value was 1.00 mg/mL against *S. aureus* (MDR). Only AcMML produced the MIC value ranging between 0.62 and 0.80 mg/mL and the MBC value ranging between 0.70 and 0.90 mg/mL against *E. coli* (MDR), *K. pneumoniae, C. albicans*, and *B. cereus*.	Maji et al. [65]
	Agar well diffusion assay	Leaves	Methanol extract	The extract was tested in the concentrations of 0.5, 1.0, 2.0, 3.0, 4.0, 6.0, 8.0, and 16.0 mg/mL	The MMML exhibited antibacterial activity at the MIC value of 3.0 mg/mL for A, B, and D and 7.0 mg/mL for C clinical strains of *S. aureus*, respectively. The MIC value recorded for the three clinical isolates of *P. aeruginosa* (A, B, and C) was 8.0 mg/mL.	
	NA	Fruits	Methanol extract	NA	The MMMFr demonstrated antibacterial activity against *B. subtilis*, *S. aureus*, *E. coli, *and *P. aeruginosa *with MIC value ranging between 62.5 and 125.0 *μ*g/mL	Koay [23]

Antiviral	*In vitro c*ytopathic effect inhibition assay	Aerial part	Methanol extract	Not appropriately described in text	The MMMAp showed moderate anti-HSV-1 activity with remarkable activity against Poliovirus. The CC_50_ value of MMMAp against HSV-1 and Poliovirus was >1000 *μ*g/mL or equal to 1000 *μ*g/mL, respectively. The EC_50_ value for MMMAp against HSV-1 virus at 20 TCID_50_ and 200 TCID_50_ was 192 and 706 *μ*g/mL. The EC_50_ value for MMMAp against Poliovirus at 20 TCID_50_ and 200 TCID_50_ was 111 and 225 *μ*g/mL, respectively.	Lohézic-Le Dévéhat et al. [32]
	Three *in vitro *methods of treatment: (i) cells (C) were inoculated with virus (V) 1 hour before treatment with extract (E), that is (C + V) + E; (ii) virus was inoculated to cells one day after treatment with extract, that is (C + E) + V; (iii) the virus and extract were added concurrently to the cells, that is C + (V + E)	Leaves	Methanol extract	Not appropriately described in text The extract was diluted at 1.0 LC_50_, 0.1 LC_50_, and 0.01LC_50_	The MMML exerted antiviral activity with different modes of action against HSV-1 or measles viruses. The MMML effectively inhibited cell death by 0.01 LC_50_ in HSV-1-inoculated cells treated using the ((C + V) + E) mode. The MMML, at 0.1 and 1.0 LC_50_, increased the cells survival from viral infection when treated using the (C + (V + E)) mode. The MMML exhibited no prophylactic effect on both test viruses when treated using the ((C + E) + V) mode	Nazlina et al. [55]

Antiparasitic	*In vivo* cotton ball-fungal mat assay	NA	Methanol extract	NA	The extract exhibited strong nematocidal activity with the recorded MED of approximately 5 mg/bl	Alen et al. [67]

Antioxidant	*In vitro *DPPH radical scavenging electron spin resonance (ESR) spectroscopic method	Flowers	Ethanol solution of crude ethyl acetate, and methanol extracts Naringenin, kaempferol and kaempferol-3*-O-*D-glucoside isolated from ethyl acetate extract Kaempferol-3*-O-*(2′′,6′′-di*-O-p-trans*-coumaroyl)-**β**-glucopyranoside and kaempferol-3*-O-*D-glucoside isolated from methanol extract	100 *μ*l (1 mg/mL) ethanol solution of the test sample was added to 100 *μ*l of DPPH (39.43 M) in ethanol solution and subjected to the assay	The MMMFw exerted a stronger free radical scavenger activity than the ethyl acetate extract. Vitamin E and Vitamin C exerted antioxidant activity higher than naringenin, kaempferol, kaempferol-3*-O-*D-glucoside, kaempferol-3*-O-*(2′′,6:-di*-O-p-trans*-coumaroyl)-**β**-glucopyranoside. The IC_50_ value for MMMFw, EAMMFw, naringenin, kaempferol, kaempferol-3*-O-*D-glucoside, kaempferol-3*-O-*(2′′,6′′-di*-O-p-trans*-coumaroyl)-**β**-glucopyranoside ranging between 6.59–35.8 *μ*g/mL.	Susanti et al. [22]
	Two *in vitro *models: (i) Ferric thiocyanate (FTC) method; (ii) 2,2-diphenyl-1-picrylhydrazyl (DPPH) (UV and ESR spectroscopic) method	Leaves	n-Hexane, ethyl acetate and methanol extract Isolated compounds (e.g., *α*-amyrin, patriscabatrine and auranamide, quercetin, quercitrin, and kaempferol-3*-O-*(2′′,6′′-di*-O-p-trans*-coumaroyl) glucosides	The exact concentration used in the FTC assay was not appropriately described. 4.0 mg of sample was mixed with a series of chemical solutions to achieved the final concentration of 0.02% w/v for the FTC assay. The concentrations of test solutions used were 500, 250, 125, 62.5, 31.3, and 7.8 *μ*g/mL in the DPPH assay	Kaempferol-3*-O-*(2′′,6′′-di*-O-p-trans*-coumaroyl) glucoside, kaempferol-3*-O-β*-D-glucose, kaempferol, hyperin, quercetin, and quercitrin showed strong antioxidative activity in the FTC method. Quercetin was found to be the most active free radical scavenger in DPPH-UV and ESR method with IC_50_ of 0.69 and 0.65 *μ*M, respectively, which was greater then vitamin E and vitamin C.	Susanti et al. [56]
	*In vitro* DPPH assay	Roots and shoots	Methanol extract	NA	The MMMR and MMMSt exhibited antioxidant activity with an IC_50_ value of 141.9 *μ*g/mL and 154.5 *μ*g/mL, respectively	Faravani [58]

Cytotoxic	*In vitro* MTT assay against two murine cancer cell lines (e.g., 3LL and L1210) and four human cancer lines (e.g., K562, U251, DU145, and MCF-7)	Aerial part	Methanol extract	Not appropriately described in text	The MMMAp demonstrated cytotoxic activity against 3LL, L1210, K562, DU145, U251, and MCF-7 with the IC_50_ value recorded ranging between 19>400 *μ*g/mL. The IC_50_ value was <25 *μ*g/mL against both murine cell lines but >25 *μ*g/mL against all human cancer cell lines.	Lohézic-Le Dévéhat et al. [32]
	*In vitro* MTT assay against MCF-7 cell line	Flowers	Ethyl acetate and methanol extracts Isolated compounds (e.g., naringenin, kaempferol and kaempferol-3*-O-*D-glucoside, kaempferol-3*-O-*(2′′,6′′-di*-O-p-trans*-coumaroyl)-**β**-glucopyranoside, and kaempferol-3*-O-*D-glucoside)	Not appropriately described in text	The 500 *μ*g/mL EAMMFw, naringenin, and kaempferol-3*-O-*(2′′,6′′-di*-O-p-trans*-coumaroyl)-**β**-glucopyranoside, but not MMMFw, exerted cytotoxic activity against MCF-7 cells line. The IC_50_ value for naringenin and kaempferol-3*-O-*(2′′,6′′-di*-O-p-trans*-coumaroyl)-**β**-glucopyranoside was 1.3 *μ*M and 0.28 *μ*M, respectively. Kaempferol-3*-O-*(2′′,6′′-di*-O-p-trans*-coumaroyl)-**β**-glucopyranoside was more effective than tamoxifen	Susanti et al. [22]
	*In vitro* MTT assay against Vero (African green monkey,* Cercopitheus aethiops *kidney cells) and L929 (mouse fibroblast) cells lines	Leaves	Methanol extract	Concentration used were not clearly explained Different concentrations of extract were used and prepared using doubling dilutions from initial stock concentration of 1000 *μ*g/mL	The MMML was not cytotoxic to both cells with LC_50_ values of 750 *μ*g/mL and >1000 *μ*g/mL, respectively	Nazlina et al. [55]

Anticoagulant	*In vitro *assay using the blood samples drawn from healthy volunteer donors (*n* = 36) of both genders (18–50 years old) The coagulation parameters measured using STA Compact coagulation analyzer were the activated partial thromboplastin time (aPTT), prothrombin time (PT), and thrombin time (TT) with cut-off time of 180 s Blood-clot-based assay with cut-off time of 300 s	Leaves	Hot water extract, cold water extract, and methanol extract	Concentrations ranging between 100 and 1000 *μ*g/mL	The 1000 *μ*g/mL hot-WMML prolonged aPTT, PT and TT in plasma but did not clot the plasma samples. The cold-WMML and MMML also prolonged aPTT, PT, but both extracts did not affect the TT. 100 to 1000 *μ*g/mL hot-WMML prolonged aPTT in a concentration-dependent manner with anticoagulant activity recorded at the concentration beyond 400 *μ*g/mL. The hot-WMML did not exhibit blood clotting effect as indicated by prolonged aPTT beyond 300 s at 900 and 1000 *μ*g/mL	Manicam et al. [68]

Platelet-activating factor (PAF) inhibitor	*In vitro *PAF binding to rabbit platelets assay	Leaves	Methanol extract	200, 100, 50, 20 and 10 *μ*g/mL	The MMML produced <10% inhibitory effect against PAF.	Jantan et al. [69]
	*In vitro *PAF binding to rabbit platelets assay	Leaves	**α**-Amyrin, betulinic acid, quercetin and quercitrin	Serial concentrationdilution range of 18.2–1.8 *μ*g/mL (18.2, 9.1, 4.5, and 1.8 *μ*g/mL)	At 18.2 *μ*g/mL, all compounds exerted inhibitory action ranging between 40 to 70% with effectiveness seen in the sequence of **α**-amyrin, betulinic acid, quercetin, and quercitrin. The IC_50_ value for **α**-amyrin, betulinic acid, quercetin, and quercitrin, ranged between 20.0 and 45.4 *μ*M	Mazura et al. [70]

Wound healing	Two types of *in vivo *wound models in rats: (i) the excision wound model; (ii) the incision wound model	Leaves	Methanol extract in the form of ointment	The extract was prepared as 5% ointment; applied topically	In the excision wound model: the MMML exhibited a wound healing activity by increasing wound contracting ability, wound closure time, tensile strength, and regeneration of tissues at the wound site; the time to wound closure of the nitrofurazone- and the MMML-treated groups was same (18 ± 2 days). In the incision wound studies: the MMML ointment increased the tensile strength of the 10-day-old wound; the MMML ointment also enhanced original tissue regeneration of the skin wounds with less fibrosis formation	Sunilson et al. [66]

Antiulcer	*In vivo *ethanol-induced gastric mucosal injuries in rats	Leaves	Aqueous extract	250 and 500 mg/kg; given orally	Macroscopically, the AMML reduced the formation of gastric mucosal injuries in a dose-dependent manner. Microscopically, the 500 mg/kg AMML provided the best protection to the gastric mucosa in rats against ethanol-induced gastric ulcers	Hussain et al. [71]

Antidiarrheal	Four *in vivo *experimental models of diarrhea in mice: (i) model 1: mice were given test solutions and fecal materials were collected for 12 h after treatment, dried in an incubator and weighed; (ii) model 2: an overnight fasted male mouse was induced with diarrhea by oral administration of castor oil (0.5 mL/mouse, p.o.) 1 hour after the test solutions administration; (iii) model 3: the overnight fasted mice were subjected to the enteropooling assay method to find out the accumulation of intestinal fluid secretion evoked by MgSO_4_; (iv) model 4: the over night fasted animals were subjected to the gastrointestinal transit test and the distance travelled by the charcoal plug from pylorus to caecum was determined andexpressed as a percentage of the total length of the small intestine	Leaves	Water extract	100, 200, and 500 mg/kg; given orally	The WMML reduced the dried fecal output of the mice (model 1). The WMML protected the mice against castor-oil-induced diarrheal droppings (model 2). The WMML dose-dependently reduced the intestinal fluid secretion induced by MgSO_4_ (model 3). The WMML inhibited the small intestinal motility of the charcoal marker in mice in a dose-dependent manner (model 4)	Sunilson et al. [61]

Antivenom	*In vitro *chick embryonic fibroblast cell lysis after *H. laoticus *scorpion venom treatment	Roots	Aqueous extracts	0.406 and 0.706 mg/mL	The AMMR only caused less than 40% antivenom efficiency at both doses tested	Uawonggul et al. [72]

Anti-inflammatory	*In vivo *carrageenan-induced paw edema in rats	Leaves	Aqueous extract	10%, 50%, and 100% strength concentration (equivalent to the doses of 4.87, 24.35, and 48.7 mg/kg); given subcutaneously	The 10–100% AMML demonstrated anti-inflammatory activity in a concentration-independent manner. The onset of anti-inflammatory action was observed 1 hour after the AMML subcutaneous administration.	Zakaria et al. [30]
	*In vivo *12*-O-*tetradecanoylphorbol-13-acetate- (TPA-) induced mouse ear oedema assay		Pure compounds obtained from *n*-hexane, ethyl acetate and methanol extracts	20 *μ*L of 0.5 mg/ear; applied topically	0.5 mg/mL kaempferol-3*-O-*(2′′,6′′-di*-O-p-trans*-coumaroyl) glucoside and *α*-amyrin demonstrated the strongest anti-inflammatory activity with the IC_50_ value of approximately 0.11 and 0.34 mM/ear, respectively. No data on the anti-inflammatory effect of crude extracts were given for comparison with their pure compounds	Susanti et al. [56]

Antinociceptive	Two *in vivo* models: (i) acetic acid-induced abdominal constriction test in mice; (ii) hot plate test in mice	Stem barks and leaves	Ethanol extract	30, 100, and 300 mg/kg; given intraperitoneally	The EMMSbl demonstrated antinociceptive activity in a dose-dependent manner in both tests. The ED_50_ recorded for the abdominal constriction test was approximately 100 mg/kg 5 mg/kg naloxone (a nonselective opioid antagonist; given intraperitoneally) inhibited the antinociceptive activity of extract in both tests	Sulaiman et al. [73]
	Three *in vivo* models: (i) acetic-acid-induced abdominal constriction test in mice; (ii) hot plate test in mice; (iii) formalin test in rats	Leaves	Aqueous extract	10%, 50%, and 100% strength concentration (equivalent to the doses of 4.87, 24.35, and 48.7 mg/kg); given subcutaneously	The AMML exerted antinociceptive activity in all three tests. In the abdominal constriction- and hot plate-test, the AMML antinociceptive activity was observed in a concentration-independent manner. In the formalin test, the AMML showed antinociceptive activity in both the early and late phases of the test with concentration-dependent activity seen only in the late phase of the test	Zakaria et al. [30]

Antipyretic	*In vivo *Brewer's yeast- (BY-) induced pyrexia test in rats	Leaves	Aqueous extract	10%, 50%, and 100% strength concentration (equivalent to the doses of 4.87, 24.35, and 48.7 mg/kg); given subcutaneously	The AMML reduced temperature of pyrexia-induced rats for the first 6 hours after BY administration	Zakaria et al. [30]
